# 2-APB and CBD-Mediated Targeting of Charged Cytotoxic Compounds Into Tumor Cells Suggests the Involvement of TRPV2 Channels

**DOI:** 10.3389/fphar.2019.01198

**Published:** 2019-10-15

**Authors:** Hagit Neumann-Raizel, Asaf Shilo, Shaya Lev, Maxim Mogilevsky, Ben Katz, David Shneor, Yoav D. Shaul, Andreas Leffler, Alberto Gabizon, Rotem Karni, Alik Honigman, Alexander M. Binshtok

**Affiliations:** ^1^Department of Medical Neurobiology, Institute for Medical Research Israel-Canada, Faculty of Medicine, The Hebrew University of Jerusalem, Jerusalem, Israel; ^2^The Edmond and Lily Safra Center for Brain Sciences, The Hebrew University, Jerusalem, Israel; ^3^Department of Biochemistry and Molecular Biology, Institute for Medical Research Israel-Canada, Faculty of Medicine, The Hebrew University of Jerusalem, Jerusalem, Israel; ^4^Department of Anesthesiology and Intensive Care Medicine, Hannover Medical School, Hannover, Germany; ^5^Shaare Zedek Medical Center and Faculty of Medicine, The Hebrew University of Jerusalem, Jerusalem, Israel

**Keywords:** TRPV2 channels, targeted delivery, hepatocellular carcinoma, BNL1 ME cells, membrane permeation, doxorubicin, cannabidiol, 2-APB

## Abstract

Targeted delivery of therapeutic compounds to particular cell types such that they only affect the target cells is of great clinical importance since it can minimize undesired side effects. For example, typical chemotherapeutic treatments used in the treatment of neoplastic disorders are cytotoxic not only to cancer cells but also to most normal cells when exposed to a critical concentration of the compound. As such, many chemotherapeutics exhibit severe side effects, often prohibiting their effective use in the treatment of cancer. Here, we describe a new means for facilitated delivery of a clinically used chemotherapy compound' doxorubicin, into hepatocellular carcinoma cell line (BNL1 ME). We demonstrate that these cells express a large pore, cation non-selective transient receptor potential (TRP) channel V2. We utilized this channel to shuttle doxorubicin into BNL1 ME cells. We show that co-application of either cannabidiol (CBD) or 2-APB, the activators of TRPV2 channels, together with doxorubicin leads to significantly higher accumulation of doxorubicin in BNL1 ME cells than in BNL1 ME cells that were exposed to doxorubicin alone. Moreover, we demonstrate that sub-effective doses of doxorubicin when co-applied with either 2-APB or CBD lead to a significant decrease in the number of living BNL1 ME cell and BNL1 ME cell colonies in comparison to application of doxorubicin alone. Finally, we demonstrate that the doxorubicin-mediated cell death is significantly more potent, requiring an order of magnitude lower dose, when co-applied with CBD than with 2-APB. We suggest that CBD may have a dual effect in promoting doxorubicin-mediated cell death by facilitating the entry of doxorubicin *via* TRPV2 channels and preventing its clearance from the cells by inhibiting P-glycoprotein ATPase transporter. Collectively, these results provide a foundation for the use of large pore cation-non selective channels as “natural” drug delivery systems for targeting specific cell types.

## Introduction

Inhibition of specific enzymes, formation of protein complexes, or modification of transcription factors' activity affects either cell state, such as excitability or activity, or even cell fate, by modulating survival, growth, division, etc. Therefore, it can be useful to alter the function of cells in diverse ways for the treatment of many different disease states. Some of the anticancer chemotherapeutics—for example, affect tumor cells by acting on enzymes that are involved in replication or uncoiling of DNA together with activation of various complex molecular signals, which ultimately induces apoptosis (Tacar et al., 2013; Dobbelstein and Moll, 2014). A limitation of this approach is that it is not cell-type-specific, resulting in damage to healthy tissue. The effect on DNA or the enzymes, or their isoforms, which are widely expressed in many cell types implies that chemotherapy also produces undesired effects. Hence, the relative effectiveness of chemotherapeutics should always be considered together with the underlying chemotherapeutic-mediated toxicity. This toxicity may affect both rapidly dividing and postmitotic non-cancer cells, thus leading to substantial side effects (Bagnyukova et al., 2010). The ultimate goal in anticancer drug development is to target only cancer cells, sparing normal cells. Several approaches are currently being used to enhance the effect of anticancer drugs on tumor cells. Some of these strategies are tuned to target cancer-specific cellular machinery. Others, by using polymeric drug carriers, liposomes, and other nanoparticles, enhance the delivery of non-specific chemotherapeutics to the tumor cells by modifying the drug tissue biodistribution (Gabizon et al., 2014). Here, we unveil a different method for the selective targeting of tumor cells. We target otherwise membrane-impermeable hydrophilic chemotherapy agents into cancer cells *via* the pore of cation non-selective transient receptor potential (TRP) channels, expressed in a differential manner by many types of tumor cells. These channels such as TRPV1, TRPV2, as well as other numerous members of TRP channel family play a critical role in tumorigenesis, tumor vascularization, and the ability of tumor cells to proliferate and migrate (Prevarskaya et al., 2007; Santoni and Farfariello, 2011; Fiorio Pla and Gkika, 2013; Chen et al., 2014). Here, we hypothesized that TRP channels could be utilized as cell-specific “natural” drug delivery system for targeting charged molecules that are cytotoxic or anti-proliferative when inside the cells, but relatively innocuous outside, specifically into cancer cells. Recently, we showed that the pore of the TRPV1 and TRPA1 channels, members of TRP channel family, which are expressed by pain- and itch-related neurons but not by other peripheral neurons, is large enough to allow passage of a charged derivative of lidocaine, QX-314. QX-314 was shown to be ineffective when applied extracellularly but blocks sodium channels and consequently neuronal excitability when it gains access to the inside of cells (Binshtok et al., 2007; Roberson et al., 2011). We have shown that activation of TRPV1 and TRPA1 channels provides a pathway for selective entry of QX-314 into pain-related (nociceptive) neurons and therefore inhibition of pain signals without effecting non-nociceptive sensory and motor neurons (Binshtok et al., 2007; Binshtok et al., 2009a; Binshtok et al., 2009b). We also have demonstrated that this approach is not limited to nociceptive neurons and could be used to selectively block other types of cells that express TRP channels (Roberson et al., 2013). We and others have suggested that this method could also be used for targeted delivery of charged cytotoxic compounds into tumor cells that express large cationic channels (Bean et al., 2007; Santoni and Farfariello, 2011; Nabissi et al., 2013). Here, we tested this hypothesis by targeting mouse hepatocellular carcinoma BNL1 ME cells with a clinically used chemotherapy drug, doxorubicin. Doxorubicin is one of the most commonly used chemotherapeutic drugs for the treatment of hepatocellular carcinoma (HCC (Bruix and Sherman, 2011) and other cancers such as lymphomas, leukemia, breast, lung, ovarian, gastric and thyroid malignancies (Lal et al., 2010). However, due to its relatively high dissociation constant (pKa), doxorubicin resides in part in its protonated, membrane impermeant form even in physiological pH (Webb et al., 2011). Considering that the tumor cell environment is of a lower than normal physiological extracellular pH (Gallagher et al., 2008; Webb et al., 2011), the protonated fraction of doxorubicin in the vicinity of tumor cells is even higher. Hence, its relative membrane impermeability is lower. Therefore, in order to increase the probability of drug permeation into tumor cells, the application of high doses is required when applying the standard therapeutic strategy. The usage of high doses, however, promotes drug off-target side effects.

Here, we show that, differently from non-cancerous liver and heart cells, mouse hepatocellular carcinoma BNL1 ME cells express a large-pore cationic channel receptor, TRPV2. Application of compounds that activates and opens TRPV2 channels facilitates the entry of doxorubicin into BNL1 ME cells, leading to its substantial accumulation within BNL1 ME cells. Moreover, we show that low sub-effective doses of doxorubicin, which do not lead to cell death, become effective and sufficient to cease proliferation and induce cell death of BNL1 ME cells when doxorubicin is co-applied with TRPV2 activators. Such CBD- or 2-APB-mediated facilitated entry will minimize the off-target effect of doxorubicin and therefore will substantially reduce adverse side effects.

## Materials and Methods

### Cell Culture

Murine BNL1 ME A.7R.1 cells (American Type Culture Collection, Manassas, VA) were plated on γ-irradiated mouse embryonic fibroblasts or 0.1% gelatin-coated six-well plates and maintained in DMEM (high glucose, Invitrogen) with 10% FBS, 2mM L-glutamine, 100 U/ml penicillin, and 100 U/ml streptomycin. Medium was changed every other day.

HEK293T cells were grown at 37°C with 5% CO_2_ in DMEM with 10% FCS and 1% penicillin-streptomycin (Biological Industries). HEK293T cells were transfected using TRPV2 cDNA and DsRed cDNA (Clontech). Transfections were performed with the TransIt (Mirus) Transfection Reagent, with equal amounts of cDNA, according to the manufacturer’s instructions and protocol.

### qPCR

Total RNA was extracted with TRI Reagent (Sigma), and 2 µg of total RNA was reverse transcribed using the M-MLV reverse transcriptase (Promega). Quantitative PCR was performed on the cDNA using SYBR Green (Roche) and the CFX96 (Bio-Rad) real-time PCR machine. TRPV2 mRNA levels were examined from mouse liver, heart tissues, and mouse HCC BNL1 ME. Samples were compared to a standard curve, which was established by serial dilutions of a known concentration of cDNA. GAPDH was used from normalization. Primers: TRPV2: 5’-TAC GGT CCT GCT CGA GTG TC-3’ and 5’-TGG CTC TAA AAC CAC CAT GC-3.’ GAPDH: 5’-CCC AGC ACA ATG AAG ATC AA-3’ and 5’-TAG AAG CAT TTG CGG TGG AC-3.’

### Immunoblotting

Cells were lysed in Laemmli buffer and analyzed for total protein concentration as described (Karni et al., 2007). Fifty micrograms of total protein from each cell lysate was separated by SDS-PAGE and transferred onto a nitrocellulose membrane. The membranes were blocked with 5% milk and probed with specific antibodies. Bands were visualized using enhanced chemiluminescence detection. Primary antibodies were as follows: RL-1 antibody (1:1,000, Santa Cruz) human origin (Caprodossi et al., 2008; Nabissi et al., 2010) and β-tubulin (1:2,000, Sigma). Secondary antibodies used were as follows: HRP-conjugated goat anti-mouse, goat anti-rabbit, and donkey anti-goat IgG (1:10,000 Jackson Laboratories).

### Ratiometric Calcium Imaging

Cultured BNL1 ME cells were loaded for 45 min with 1 μM FURA-2 AM dye (stock in DMSO) in a standard external solution (SES) composed of (in mM) 5 KCl, 145 NaCl, 2 CsCl, 1 MgCl, 10 HEPES, and 10 glucose and then rinsed for 45 min for de-esterification of intracellular acetoxymethyl esters. Cells were perfused continuously at a rate of ∼1 ml per min with SES and examined with an inverted microscope equipped with Epi-Fl attachment, perfect focus system (Nikon) and EXi Aqua monochromator (Q-imaging) and X40 lens. Intracellular Ca^2+^ concentrations were measured fluorometrically as the absorbance ratio at 340 and 380 nm (Δ*F*340/380, 510 nm for emission, Lambda DG4, Sutter Instruments). Images were taken every 1 s, monitored online, and analyzed offline using Nikon Elements AR Software (Nikon). 2-Aminoethoxydiphenyl borate (2-APB, 200 μM) or cannabidiol (CBD, 10 μM) were briefly bath applied (as indicated in the figures) using a fast-step valve control perfusion system. In some experiments, CBD was applied after cells were pre-treated with 1 µM doxorubicin. For a positive control of imaging, an ionophore, ionomycin (1 µM, Sigma), was briefly bath applied, at the end of the experiment. In some experiments, cells were treated with 1 µM thapsigargin for 5–15 min, in order to deplete endoplasmic reticulum Ca^2+^ stores (Thastrup et al., 1990). We considered the cells as responsive only if the changes in ratio (Δ*F*) following application of 2-APB (200 μM) or CBD (10 μM) were larger than 0.1Δ*F*, and were easily distinguishable from optic noise which was about 0.02 Δ*F*.

### Doxorubicin Imaging

Cultured BNL1 ME cells were perfused continuously at 2 ml per min with DMEM and examined with an inverted microscope equipped with Epi-Fl attachment, perfect focus system (Nikon) and EXi Aqua monochromator (QImaging). Doxorubicin fluorescence was measured as absorbance at 480 nm (580 nm for emission, Lambda DG4, Sutter Instruments). Images were taken every 10 min, monitored online, and analyzed offline using Nikon Elements AR Software (Nikon). 2-APB (200 μM) or CBD (10 μM) and doxorubicin (1 and 5 µM) were bath applied using a fast-step valve control perfusion system, at a rate of ∼1 ml per min.

### *In Vitro* Cell Growth Assay

This assay was carried out as previously described (Shneor et al., 2017). Briefly, BNL1 ME cells were plated in 96-well plates for 24 h. Then, each one of the treatment groups was added. Dose responses of doxorubicin and each one of the activators were used to find the optimal doses for the drugs. Cells were then incubated with DMEM with one of the drugs for 24, 48, and 72 h. Then, cells were incubated for 30 min with substrate of CellTiter-Fluor Cell Viability Assay (Promega; excitation: 380–400 nm; emission 505 nm). Live cells took up the substrate, and constitutive protease activity cleaved it to the fluorescent form, which generated a fluorescent signal proportional to the number of live cells. The fluorescent signal was detected in a flow cytometer.

### Clonogenic Cell Survival Method

BNL1 ME cell populations were prepared by trypsinization. Cells were counted using a hemocytometer, and appropriate cell numbers were seeded in six-well plates and treated for 24 h with doxorubicin alone or together with TRPV2 activators. Colonies were fixed and stained with methylene blue (Karni et al., 1999; Karni et al., 2007); for quantification, the number of colonies in each well was calculated using a stereomicroscope. Digital images of the colonies were obtained using a CCD camera (Nikon, Japan). Colonies were counted using ImageJ analysis software (Fiji version 1.44a). Due to the high confluence of the colonies after the treatment with DMSO or CBD alone, the examined parameters of the number of colonies, the size of the individual colony, and the inner density of each colony were undetectable. Treatment with 2 µM doxorubicin alone completely prevented colony formation. Therefore, only colonies following treatment with doxorubicin and colonies treated with both CBD and doxorubicin were analyzed and compared.

### Doxorubicin Uptake Assay

Intracellular doxorubicin concentrations were measured with or without TRPV2 activators. BNL1 ME cells were plated on six-well plates at concentrations of 2 × 10^5^ cells/well and treated with DMEM for 24 h, after incubation at 37°C for 1 h. With each one of the treatments, each culture medium was removed, and cells were washed three times with PBS. The cells were lysed in 1 ml HCL-acidified isopropanol for 24 h (centrifuged at 2,000 rpm for 10 min). For fluorometric analysis, total cellular doxorubicin in BNL1 ME cells was determined by measuring the fluorescent emission of the solution (λ_ex_ = 480 nm, λ_em_ = 590 nm) in the cell lysate with a fluorometer, as described elsewhere (Patil et al., 2018). The drug concentration was calculated with the standard curve of doxorubicin.

### Calcein Accumulation Assay

BNL1 ME cells were seeded at 20 × 104 cells/well in 24-well plates. Experiments were performed 2 days after achieving confluent monolayers. Before the experiment, cells were incubated for 1 h with 1 ml DMEM, supplemented with 5 mM HEPES, pH 7.3, in the presence or the absence of a P-gp inhibitor, verapamil (200 µM). In the accumulation phase, cells were co-incubated with 0.25 µM calcein-AM in the presence or absence of the inhibitor. Control cells were incubated with equal volumes of solvents of all substances (DMSO). After 1 h, the cells were washed three times with ice-cold PBS. Intracellular fluorescence of calcein-AM was measured within 1 h with λ_ex_ = 485 nm and λ_em_ = 528 nm using a plate reader (Synergy HT, BioTek, Winooski, VT, USA).

### Chemicals

Doxorubicin and 2-APB were purchased from Sigma. CBD was kindly provided by the lab of Prof. Raphael Mechoulam, School of Pharmacy, Hebrew University of Jerusalem, Hadassah Ein Kerem. Calcein was kindly provided by the lab of Dr. Sara Eyal, School of Pharmacy, Hebrew University of Jerusalem, Hadassah Ein Kerem.

### Experimental Design and Statistical Analysis

Data are shown as mean ± S.E.M. Differences between groups were analyzed using a two-tailed Student’s *t*-test or one-way ANOVA analysis of variance followed by Bonferroni *post hoc* tests, when appropriate. The criterion for statistical significance was *p* < 0.05.

Sample size calculation: we did not carry out a power analysis because we were studying the effect of a new drug combination and had no way to estimate the effect size.

The number of replicates (n for cells and n for the number of plates/repetitions) for each experiment is given either in the Figure legends or in the *Results*. For each treatment group, we calculated the average value for the repetitions and ran the statistical comparisons between the treatments. If a representative example is shown, we explain how representative it is, i.e., how many cells/plates showed a similar effect. When relevant, the inclusion criteria for the experiments are described in the *Materials and Methods* section above.

## Results

### Mouse Hepatocellular Carcinoma BNL1 ME Cells Express Functional TRPV2 Channels

**Figure 1:**
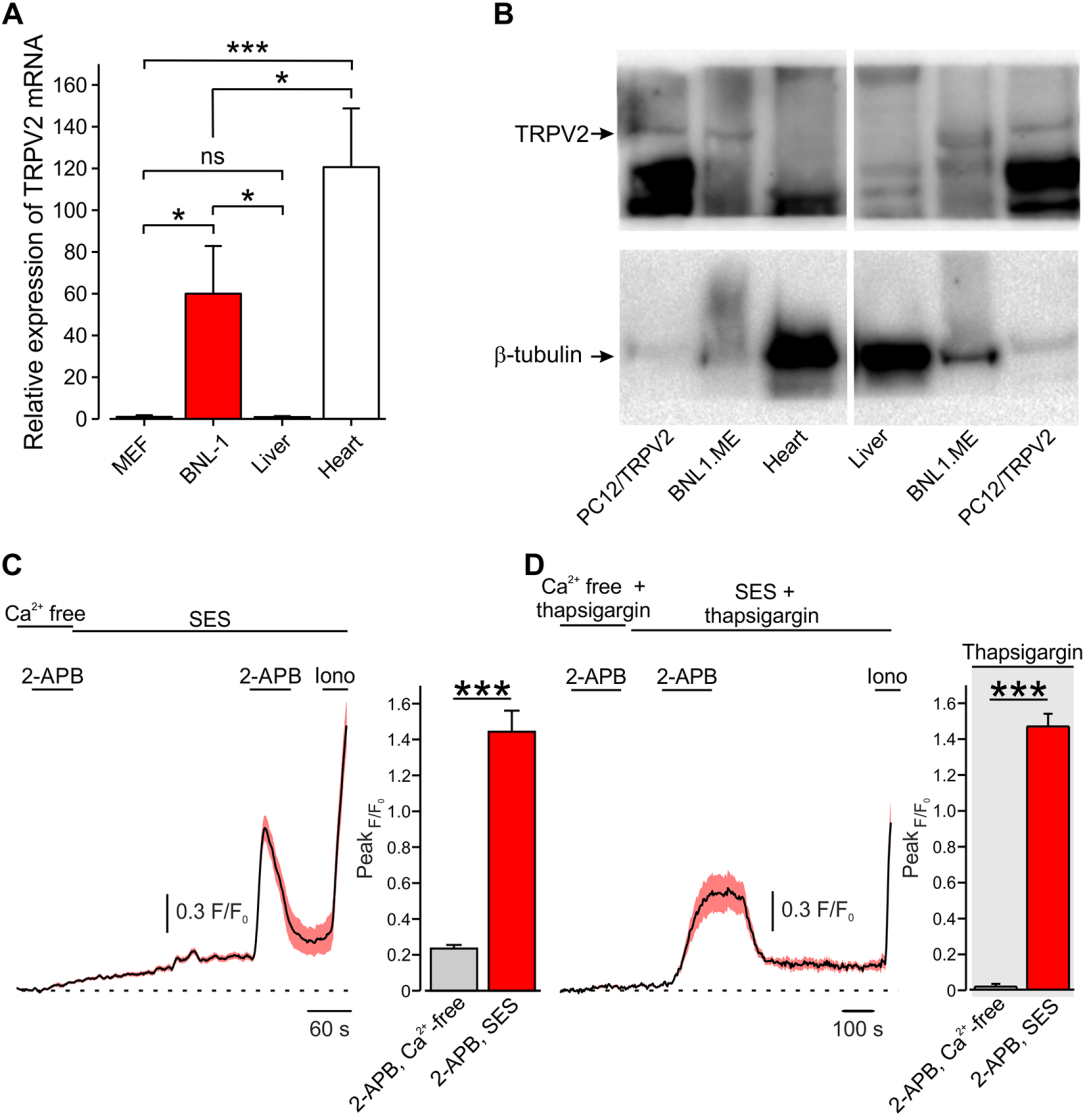
Mouse hepatocellular carcinoma BNL1 ME cells, but not liver or heart cells express functional TRPV2 channels. **(A)** Bar graph depicting relative expression levels, assessed by RT-PCR, of TRPV2 mRNA in BNL1 ME cells compared to MEF, non-cancerous mice liver, and mice heart cells (GAPDH was used for normalization; *see Methods*). *—p < 0.05; ***—p < 0.001, one-way ANOVA with *post hoc* Bonferroni, n = 3 replications. **(B)** Western blot analysis of TRPV2 protein levels in BNL1 ME cells compared to pheochromocytoma 12 cell line (PC12) constitutively expressing TRPV2 (PC12/TRPV2), non-cancerous mice liver cells, and mice heart cells. β-Tubulin served as a control (for a blot containing all the lanes; see **Supplementary Figure 1**). The size of the proteins (95 kD for TRPV2 and 51 kD for β-tubulin) was determined relative to pre-stained size markers. Representative of five experiments. **(C)** *Left*, Mean ± SEM of changes in cytosolic [Ca^2+^]_i_ following bath application of 200 μM 2-APB. Note that application of 2-APB leads to a small increase in intracellular Ca^2+^ when external solution contains nominal Ca^2+^ concentration (Ca^2+^ free) but produces a substantial and significant (***—p < 0.001, one-way ANOVA) increase in intracellular Ca^2+^ when calcium is present in the bath solution (standard external solution, SES), n = 26 cells. For a positive control of imaging, 1 µM ionomycin (Iono) was added to the bath solution at the end of each experiment. *Right*, bar graph comparing peak changes in cytosolic [Ca^2+^]_i_ following application of 2-APB in Ca^2+^ free-solution with changes in cytosolic [Ca^2+^]_i_ following application of 2-APB in 2 mM Ca^2+^ containing SES; ***—p < 0.001, paired Student *t*-test, n = 43 cells, from three plates. **(D)** Same as in *C*, but with SES containing thapsigargin. Note that, under these conditions, the increase in intracellular Ca^2+^ occurs only when calcium is present in the external solution (SES) n = 28 cells. *Right*, ***—p < 0.001, paired Student *t*-test, n = 34 cells, from three plates. ns, not significant.

Amongst the large pore-cation non-selective ion channels, the TRP vanilloid subtype 2 channel (TRPV2), a member of the TRP superfamily, is highly expressed by a variety of tumor cells (Santoni and Farfariello, 2011; Liberati et al., 2014). It has been shown that urothelial carcinoma cells (Caprodossi et al., 2008; Mizuno et al., 2014), human leukemic cells (Pottosin et al., 2015), prostate cancer cells (Monet et al., 2010), esophagus squamous cell carcinoma cells (Zhou et al., 2014), and hepatocellular carcinoma cells (Liu et al., 2010) overexpress TRPV2 channels. Here, we sought to examine whether a pore of TRPV2 channels expressed by a murine model of hepatocellular carcinoma cells [BNL1 ME (Kuriyama et al., 1999; Tatsumi et al., 1999; Ogunwobi and Liu, 2011)] could be used to shuttle doxorubicin into these cells selectively. First, we examined whether BNL1 ME cells overexpress TRPV2 similarly to previous reports of hepatocellular carcinoma cells (Liu et al., 2010). We compared the expression levels of TRPV2 in BNL1 ME cells to a mouse embryonic fibroblast cell line (MEF), which served as a negative control, and to normal mouse liver cells from a direct, first passage culture. We used RT-qPCR and showed that BNL1 ME cells possess significantly higher levels of TRPV2 mRNA than MEF and normal liver cells (**Figure 1A**). We next compared the protein level of TRPV2 channel in BNL1 ME, dissociated mouse liver cells, and pheochromocytoma 12 cell line (PC12) transfected with TRPV2 as a positive control (**Figure 1B**). We demonstrated that, while the levels of the TRPV2 protein are undetectable in liver cells, BNL1 ME cells express substantial levels of TRPV2.

Considering that cardiotoxicity is one of the significant side effects of doxorubicin (Singal and Iliskovic, 1998; Chatterjee et al., 2010) and that expression of TRPV2 was previously shown in a variety of heart cells e.g., cardiomyocytes, fibroblasts, endothelial cells, and vascular smooth muscle cells (Watanabe et al., 2009), we also compared the levels of TRPV2 mRNA and TRPV2 channel protein in short-term cultured heart cells from mouse heart to BNL1 ME cells. Indeed, we confirmed that heart cells express TRPV2 mRNA (**Figure 1A**); however, these cells did not show expression of the TRPV2 protein (**Figure 1B**). This inconsistency implies posttranslational modulation, which is a common mechanism to strictly regulate protein levels (Maier et al., 2009; Vandewauw et al., 2013). This posttranslational regulation is also true for TRPV2 regulation (Uhlen et al., 2015). These data indicate that mouse BNL1 ME cells express TRPV2 channel protein, unlike liver or heart cells.

To assess whether TRPV2 channels expressed by BNL1 ME cells are functional, we examined changes in intracellular Ca^2+^ [(Ca^2+^)_i_] in BNL1 ME cells loaded with the Ca^2+^ indicator FURA-2AM, following bath application of the TRPV2 channel activator, 2-aminoethoxydiphenyl borate [2-APB (Hu et al., 2004; Juvin et al., 2007)]. We first perfused the cells with a bath solution containing nominally free Ca^2+^ (Ca^2+^—free bath solution) in order to examine possible 2-ABP-mediated release of Ca^2+^ from internal stores, as previously shown (Maruyama et al., 1997). In these conditions, bath application of 2-APB produced a small increase in [Ca^2+^]_i_ (**Figure 1C**). Reperfusion of cells with bath solution containing 2 mM Ca^2+^ (SES) led to a slow, gradual increase in [Ca^2+^]_i_ reaching a plateau after about 4 min, as expected. The second application of 2-APB in these conditions led to a substantial increase in [Ca^2+^]_i_ in all monitored cells (**Figure 1C**). These data suggest that 2-APB produces a large transmembrane Ca^2+^ influx into BNL1 ME cells, in addition to a small 2-APB-mediated increase, possibly *via* 2-ABP-mediated activation of IP_3_ receptors (Maruyama et al., 1997). To examine the latter possibility, we treated BNL1 ME cells for 5–15 min with 1 µM thapsigargin to deplete endoplasmic reticulum Ca^2+^ stores (Thastrup et al., 1990). 2-APB applied on thapsigargin-treated cells, perfused with Ca^2+^—free bath solution did not produce any change in [Ca^2+^]_i_. Changing the bath to a solution containing 2 mM Ca^2+^ did not result in the rise of intracellular Ca^2+^. However, the second application of 2-APB in cells treated with bath solution containing 2 mM Ca^2+^ led to a substantial increase in [Ca^2+^]_i_ (**Figure 1D**), suggesting that the 2-APB-induced [Ca^2+^]_i_ rise is due to TRPV2 and not store-operated channels. Collectively, these data suggest that 2-APB induces a transmembrane Ca^2+^ influx, indicating that BNL1 ME express functional TRPV2 channels on their plasma membrane.

To further study the functionality of TRPV2 channels, we measured changes in [Ca^2+^]_i_ in BNL1 ME cells following bath application of another well-established TRPV2 activator, cannabidiol (CBD, (Qin et al., 2008). It was previously demonstrated that CBD leads to [Ca^2+^]_i_ increase, mainly due to TRPV2-mediated Ca^2+^ influx, since CBD-mediated Ca^2+^ transients *via* TRPV2 were abolished when extracellular Ca^2+^ was removed from the external solution (Eubler et al., 2018). First, we showed that 10 µM CBD applied onto HEK293T cells, transfected with human TRPV2 (hTRPV2) led to an increase in [Ca^2+^]_i_ only in the TRPV2 transfected HEK cells, which were labeled with DsRed-expressing fluorescent protein (**Supplementary Figure 2**). Application of CBD on HEK293T cells, which were not labeled with DsRed, and therefore do not express hTRPV2, did not produce a change in [Ca^2+^]_i_ (n = 19 cells, *data not shown*). Bath applications of 10 µM CBD onto BNL1 ME cells, similarly to the effect of 2-APB, led to a robust increase in [Ca^2+^]_i_ (**Supplementary Figure 3**), further supporting the functional expression of TRPV2 on the plasma membrane of BNL1 ME cells.

### 2-APB- or CBD-Mediated Activation of TRPV2 Channels Is Sufficient to Produce Entry and Accumulation of Doxorubicin Into BNL1 ME Cells

**Figure 2:**
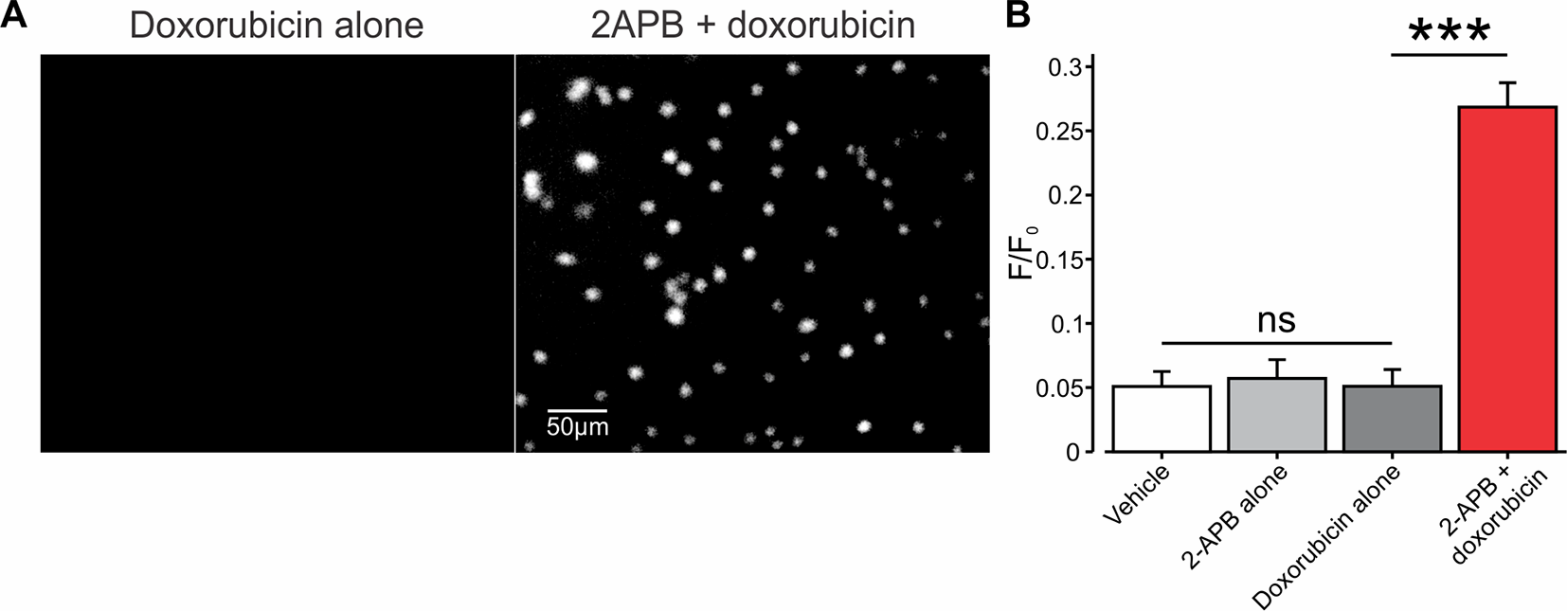
Co-application of 2-APB with doxorubicin leads to facilitated entry of doxorubicin into mouse BNL1 ME cells. **(A)** Representative epifluorescent images of cultured BNL1 ME cells, 5 min after application 1 µM doxorubicin alone (*left*) and co-application of 200 μM 2-APB with 1 µM doxorubicin (*right*). Note that doxorubicin-based fluorescence (*see Methods*) was detected only when co-applied with 2-APB. **(B)** Bar graph depicting means ± SEM of changes in intensity of intracellular fluorescence following application of vehicle, 2-APB alone, doxorubicin alone, or doxorubicin together with 2-APB at the indicated concentrations. The values for vehicle and 2-APB alone were measured 30 min after application. 2-APB was then washed out for 30 min, and the values for doxorubicin alone or doxorubicin together with 2-APB were measured after 60 min each. Note that doxorubicin-based intracellular fluorescence was above the noise levels (0.05 F/F_0_) only after the co-application of doxorubicin with 2-APB. ***p < 0.001, compared to doxorubicin alone, paired *t*-test, ns, not significant, n = 60 cells, three repetitions.

In developing our platform for facilitated entry of charged compounds into cancer cells, through TRP channels, we used the relatively small (543 Da) chemotherapeutic drug, doxorubicin, which in physiological pH resides mostly in the charged form (Webb et al., 2011). We first examined whether activation of TRPV2 channels is sufficient to allow the entry of doxorubicin into BNL1 ME cells. To that end, we exploited the endogenous fluorescence property of doxorubicin (Scalori et al., 1988) and measured the intensity of its intracellular fluorescence, that reflects doxorubicin “trapped” in the cytoplasm. To ensure that the fluorescence originates from doxorubicin which is “trapped” inside the cell and does not originate from doxorubicin in the extracellular solution, we performed the measurements of doxorubicin-induced intracellular fluorescence after 1 h of doxorubicin washout. This time frame is sufficient to allow an exchange of all extracellular solution about 50 times, suggesting that all extracellular doxorubicin is washed out. In these conditions, bath application (*see Methods*) of either 1 µM doxorubicin alone (**Figures 2A**, *left*, **B**) or 200 µM 2-APB alone (**Figure 2B**) did not cause any changes in the fluorescent levels, beyond basal control levels (**Figure 2B**). However, the co-application of doxorubicin, together with 2-APB, led to a significant increase in intracellular fluorescence (**Figures 2A**, *right*, **B**), implying that doxorubicin enters the cells only in conjunction with the activation of TRPV2.

**Figure 3:**
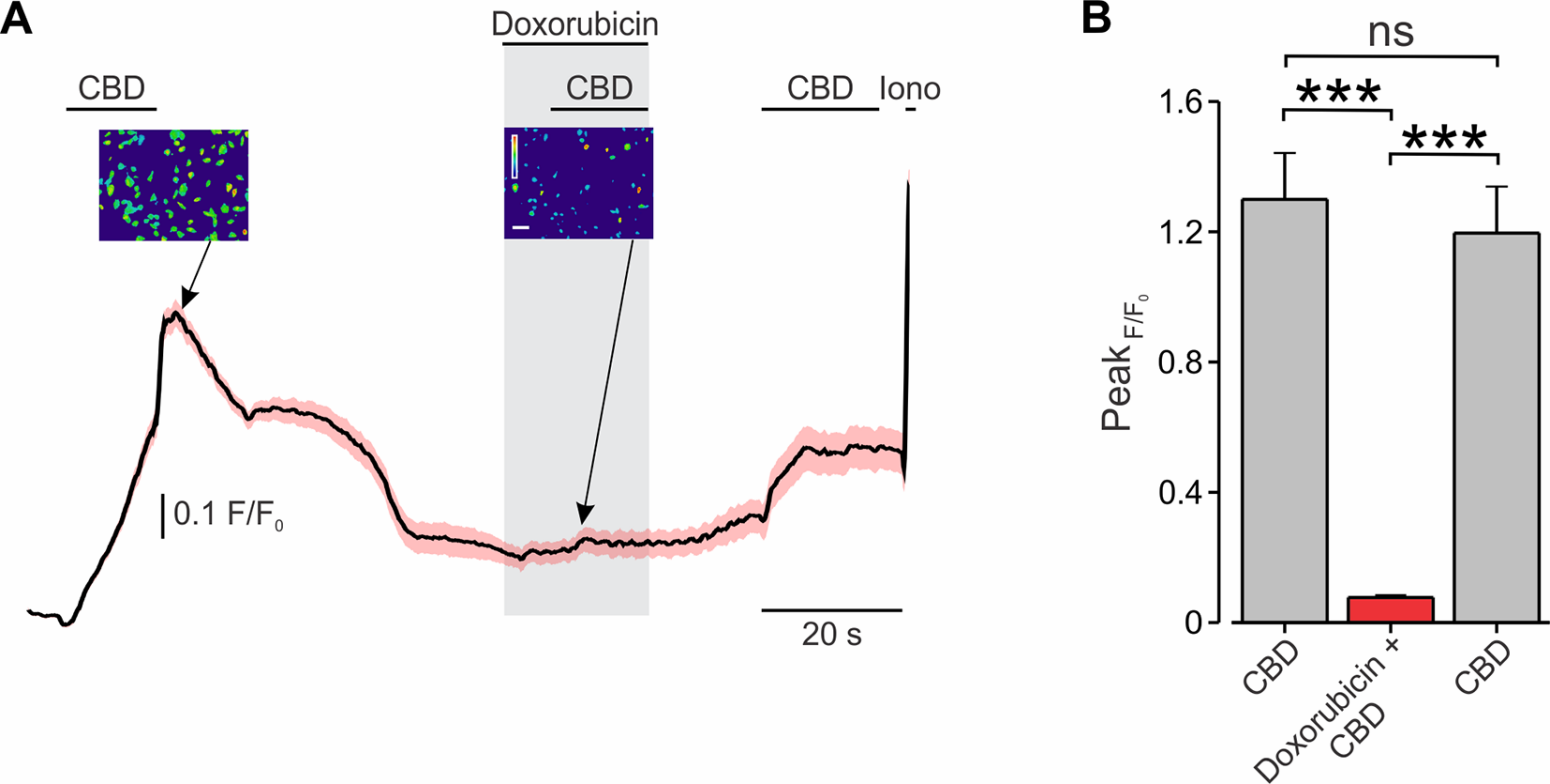
Co-application of CBD with doxorubicin attenuates TRPV2-mediated Ca^2+^ entry into BNL1 ME cells. **(A)** Mean ± SEM of changes in cytosolic [Ca^2+^]_i_ following bath application of 10 μM CBD in SES, 10 μM CBD in SES containing 1 μM doxorubicin, and 10 μM CBD in after washout of doxorubicin, n = 36 cells. *Insets*, photomicrographs of blue-green-red pseudocolor radiometry images (taken at the time points indicated by arrows) demonstrated a decrease of F/F_0_ after treatment of doxorubicin. Bar, 50 µm. The pseudocolor scale depicts changes in F/F_0_ between 0 (*blue*) to 1.5 (*red*). For a positive control of imaging, 1 µM ionomycin (Iono) was added to the bath solution at the end of each experiment. **(B)** Bar graph depicting mean ± SEM of changes in peak FURA-2AM fluorescence following bath application of 10 μM CBD in SES, 10 μM CBD in SES containing 1 μM doxorubicin, and 10 μM CBD in after washout of doxorubicin. Note a significant decrease in response to CBD when doxorubicin is present in the extracellular solution. Note also that the response to CBD is partially rescued after doxorubicin is washed out. ***—p < 0.001, ns, not significant; RM one-way ANOVA with *post hoc* Bonferroni, n = 54 cells, from three plates.

Next, we examined whether CBD-mediated activation of TRPV2 is also sufficient to shuttle doxorubicin into BNL1 ME cells. The previously described method of measuring doxorubicin fluorescence following treatment with CBD could be inappropriate, due to CBD-mediated inhibition of the P-glycoprotein ATPase transporter (Zhu et al., 2006). This transporter has been shown to participate in the removal of doxorubicin from the cells (Giavazzi et al., 1984; Ferry, 1998) and appears to be present in BNL1 ME cells (*see below and* **Supplementary Figure 6**). Thus, increased fluorescence following the application of doxorubicin and CBD could result not only because of facilitated entry of doxorubicin but also due to its accumulation following the inhibition of the transporter. We, therefore, examined the CBD-mediated doxorubicin entry into BNL1 ME cells by measuring changes in the Ca^2+^ fluxes following the application of doxorubicin together with CBD. We hypothesized that, if doxorubicin permeates through the pore of TRPV2 channels, it would impair the penetration of Ca^2+^ and therefore would lead to a decrease in the FURA2-induced fluorescence, similarly to other charged molecules such as QX-314 (Puopolo et al., 2013). Accordingly, we loaded BNL1 ME cells with FURA-2AM and compared CBD-induced increase in [Ca^2+^]_i_ in cells treated with standard bath solution (SES; *see Methods*) with cells treated with bath solution containing doxorubicin. When cells were perfused with SES, the application of 10 µM CBD produced a substantial increase in [Ca^2+^]_i_ in all cells (**Figure 3**; see also **Supplementary Figure 3**). Application of CBD onto cells treated with SES containing 1 μM doxorubicin led to significantly smaller increase in [Ca^2+^]_i_ (p < 0.001, n = 30 cells; **Figure 3**). When the doxorubicin-containing bath solution was washed out, CBD-mediated increase in [Ca^2+^]_i_ partially recovered (**Figure 3**). It is noteworthy that, when cells were perfused with SES, consecutive applications of CBD produced a similar increase in [Ca^2+^]_i_ (p > 0.05, n = 36 cells; **Supplementary Figure 3**).

Because CBD has been shown to activate other Ca^2+^ permeable ion channels (Bisogno et al., 2001), we examined whether TRPV2 channels specifically provide a pathway for the doxorubicin to enter the cells following CBD-mediated activation. To that end, we measured how doxorubicin, when added to SES, changes CBD-mediated Ca^2+^ influx into HEK293T cells, transfected with human TRPV2 (hTRPV2). Similar to our results from BNL1 ME cells, the addition of doxorubicin to the SES leads to a significant decrease in CBD-mediated Ca^2+^ influx (**Supplementary Figure 4A**). When doxorubicin was not added to the SES, subsequent applications of CBD produced similar increases in [Ca^2+^]_i_ (**Supplementary Figure 4B**). These results from the minimally reconstituted system of TRPV2-expressing HEK cells indicate that CBD-activated TRPV2 channels is sufficient to shuttle doxorubicin into the cells.

Collectively, these data imply that doxorubicin enters BNL1 ME cells *via* active TRPV2 channels. Importantly, these data also suggest that doxorubicin enters BNL1 ME cells *via* the pore of TRPV2 channels.

**Figure 4:**
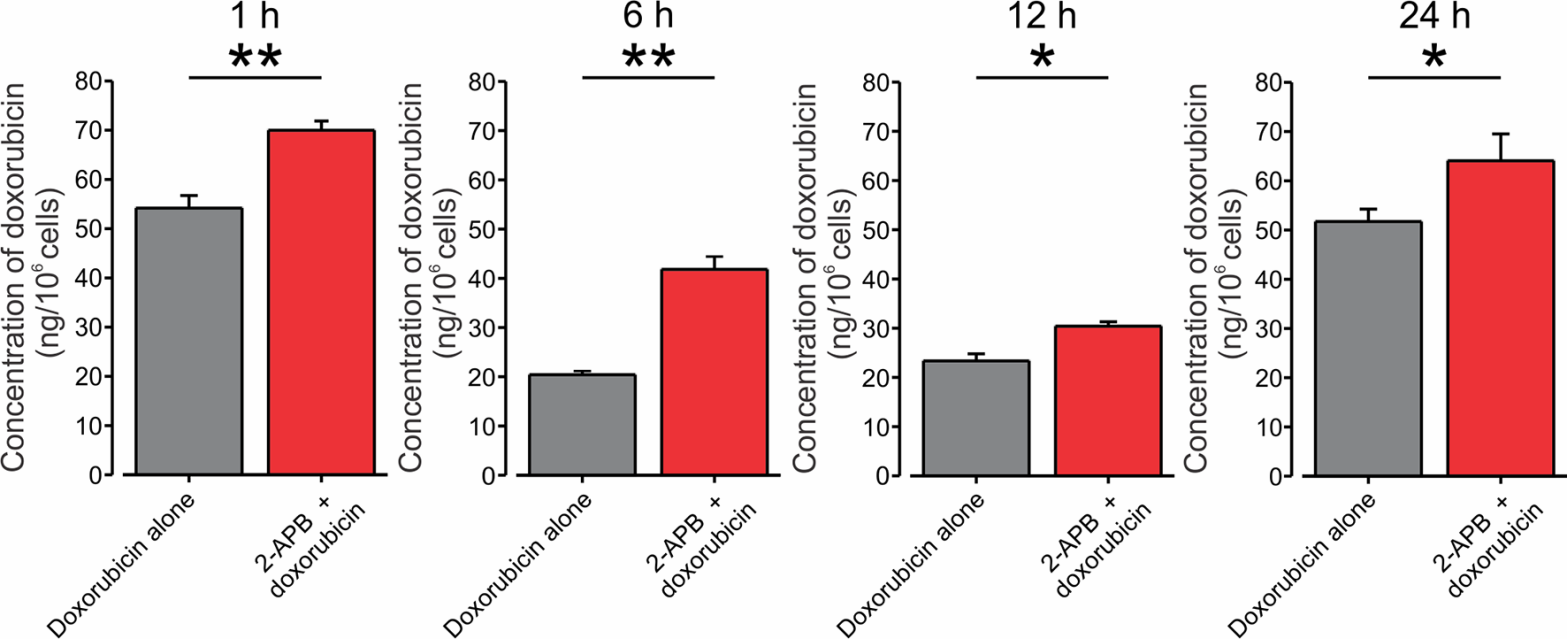
Co-application of doxorubicin with 2-APB leads to an increased amount of intracellular doxorubicin in BNL1 ME cells. Bar graphs depicting the amount of doxorubicin in BNL1 ME cells (in ng/10^6^ cells) measured using fluorometric analysis, 1, 6, 12, and 24 h following treatment with 1 µM doxorubicin alone (*gray*) or with 1 µM doxorubicin co-applied with 200 µM 2-APB (*red*). Note that the amount of intracellular doxorubicin was significantly increased when doxorubicin was co-applied together with 2-APB, *—p < 0.05, **—p < 0.01, Student’s *t*-test, n = 3 repetitions for 1-, 6-, and 12-h time points, n = 2 repetitions for 24-h time point, n = 3 repetitions, for each treatment group.

Finally, to examine directly whether activation of TRPV2 channels would lead to increased accumulation of doxorubicin in BNL1 ME cells, we measured the intracellular concentration of doxorubicin. We used an *in vitro* uptake method in which intracellular doxorubicin concentrations were measured in BNL1 ME cells by fluorometric analysis. We assessed the intracellular concentration of doxorubicin at different time points after treating BNL1 ME cells with 1 µM doxorubicin alone or together with activation of the TRPV2 channels. To prevent the possible effect of enhanced doxorubicin accumulation caused by CBD-mediated inhibition of P-glycoprotein ATPase transporter on doxorubicin concentrations, we activated TRPV2 channels using 200 μM 2-APB. We found that at all measured time points, the total cellular concentration of doxorubicin in BNL1 ME cells, was significantly higher when doxorubicin was co-applied with 2-APB than when doxorubicin was applied alone (**Figure 4**). These data suggest that activation of TRPV2 channels enhances the accumulation of doxorubicin in BNL1 ME cells.

### Activation of TRPV2 by 2-APB Leads to Facilitated Entry of Doxorubicin Into BNL1 ME Cells

**Figure 5:**
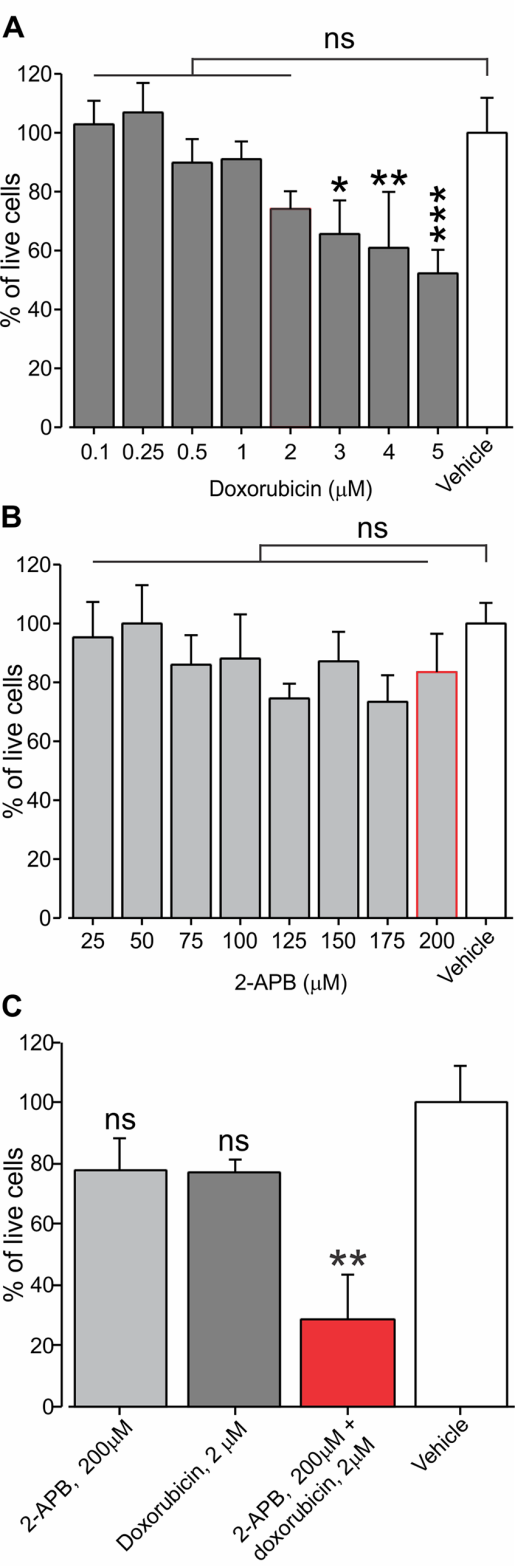
Application of sub-effective dose of doxorubicin together with 2-APB leads to a decrease in living BNL1 ME cells. **(A)** Live-cell fluorescence measurements from cultured BNL1 ME cells 24 h after treatment with doxorubicin alone at the indicated doses. Note that only treatment with doses higher than 2 µM leads to a significant reduction in the number of live cells (measured as a relative level of fluorescence; *see Methods*). **(B)** Same as in *A* but cells were treated for 24 h with 2-APB at the indicated dosed. Note that 2-APB does not affect cell viability at all examined doses. **(C)** Live-cell fluorescence measurements from cultured BNL1 ME cells show 24-h treatment with previously ineffective 2 µM doxorubicin applied together with 200 µM 2-APB leads to a significant decrease tumor cells viability, *p < 0.05, **p < 0.01, ***p < 0.001, ns, not significant; one-way ANOVA, comparison between the vehicles to all other groups, n = 6 wells for each group, three repetitions.

We hypothesized that activation of TRPV2 by virtue of opening aqueous pores, which are permeable for the protonated fraction of doxorubicin, will facilitate the permeation of doxorubicin and therefore augment its cytotoxic effect on TRPV2-expressing cancer cells, while sparing other cell types and therefore minimizing off-target side effects, as the effective concentration of doxorubicin can be lowered. To examine this hypothesis, we used a cell survival assay that measures cell viability expressed by the level of fluorescence (*see Methods*). We first determined a concentration range at which the application of doxorubicin alone does not affect the viability of BNL1 ME cells. We show here that 24-h treatment of BNL1 ME cells at concentrations of up to 2 µM doxorubicin do not lead to significant cell death (**Figure 5A**). Treatment with higher than 2 µM doxorubicin led to a significant decrease in the number of live cells (**Figure 5A**). We then chose the maximal sub-effective concentration of doxorubicin (2 µM) and co-applied it with 2-APB, which by itself did not affect cell viability in a concentration range of 25 to 200 µM, when treated for 24 h (**Figure 5B**). Co-application of 2 µM doxorubicin with 200 µM 2-APB led to a significant decrease in cell viability after 24 h (**Figures 5C, D**). The observed effect of co-application of doxorubicin and 2-APB showed a ∼70% decrease after 24 h. This decrease was higher than the additive effect of the two treatments (∼ 40% after 24 h, **Figure 5D**). These results suggest that activation of the TRPV2 channels leads to facilitated entry of doxorubicin into BNL1 ME cells such that previously sub-effective doses of doxorubicin alone when co-applied with the activator of the TRPV2 channels are sufficient to significantly affect cell viability. At these concentrations, at the later time points of 48 and 72 h, both doxorubicin and 2-APB applied alone had a substantial effect on the number of living cells such that there was no facilitated effect of co-application of 2-APB and doxorubicin (**Supplementary Figures 5A, B**). These data show that the optimal effect of 200 µM 2-APB-mediated facilitated entry of 2 µM doxorubicin on cell vitality is achieved 24 h after initiation of treatment.

### CBD Facilitates Both the Entry and Accumulation of Doxorubicin in BNL1 ME Cells

**Figure 6:**
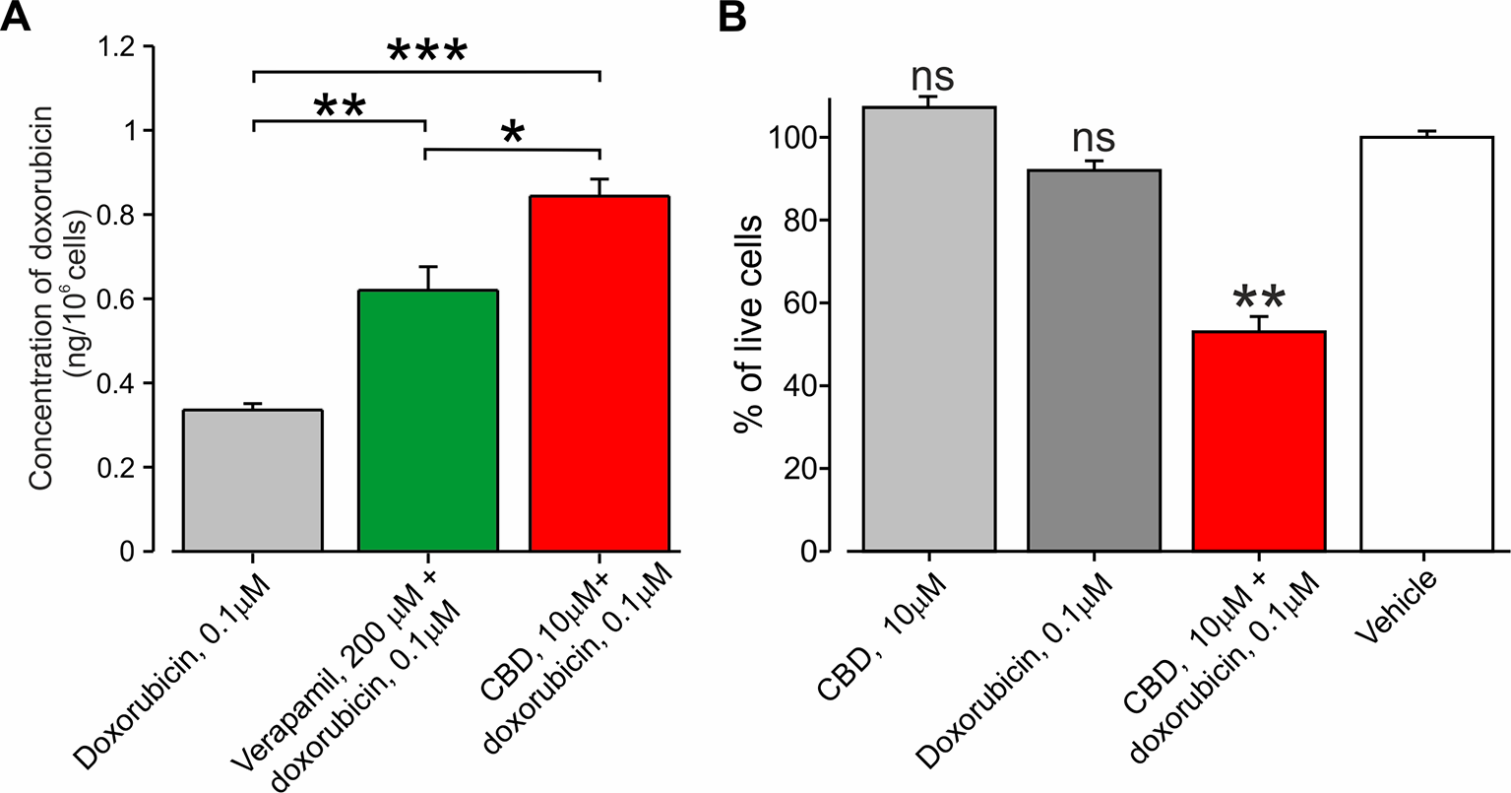
Co-application of doxorubicin with CBD led to an increased amount of intracellular doxorubicin and increased cell death. **(A)** The concentration of doxorubicin in BNL1 ME cells (in ng/10^6^ cells; *see Methods*) following a 24-h treatment with 0.1 μM doxorubicin alone, 0.1 μM doxorubicin together with 200 µM verapamil, or 0.1 μM doxorubicin together with 10 μM CBD, measured using fluorometric analysis; n = 3 repetitions for each time point for each treatment group. **(B)** Live-cell fluorescence measurements from cultured BNL1 ME cells treated for 24 h with 10 μM CBD alone, 0.1 μM doxorubicin alone or co-application of 10 μM. CBD, and 0.1 μM doxorubicin. Note that only the co-application of CBD with doxorubicin produced a significant decrease in the number of living cells. *p < 0.05, **p < 0.01, ***p < 0.001, ns, not significant; one-way ANOVA, comparison between the vehicle to all other groups, n = 6 wells for each group, three repetitions.

CBD has been shown to inhibit the P-glycoprotein ATPase transporter (Zhu et al., 2006), which participates in the removal of doxorubicin from the cells (Giavazzi et al., 1984; Ferry, 1998). We therefore hypothesize that CBD may amplify the efficacy of the doxorubicin-mediated effect specifically in cancer cells by (1) providing selective entry of doxorubicin into cancer cells *via* TRPV2 activation (see **Figure 3**) and by (2) enhancing doxorubicin accumulation by P-gp ATPase inhibition. To examine the latter notion, we examined whether BNL1 ME cells express functional P-gp ATPase by measuring the accumulation of the P-gp ATPase substrate, calcein, (Hollo et al., 1994) in BNL1 ME cells following application of the well-established P-gp antagonist, verapamil (Cornwell et al., 1987). We show that the application of verapamil significantly increases the intracellular concentration of calcein (**Supplementary Figure 6**), suggesting that BNL1 ME cells express functional P-gp ATPase. Accordingly, the co-application of verapamil and doxorubicin significantly increases intracellular doxorubicin concentrations, compared with doxorubicin alone (**Figure 6A**). Notably, doxorubicin accumulation following co-application with CBD was significantly higher than the accumulation following co-application of verapamil with doxorubicin (**Figure 6A**), implying that, in addition to a possible effect of CBD on P-gp ATPase, CBD facilitates doxorubicin accumulation by other mechanisms, possibly, as our data suggest, by promoting its entry *via* TRPV2 channels. These results also suggest that CBD co-applied with doxorubicin may be beneficial, compared to 2-APB, in causing cell death. Indeed, we were able to achieve about 40% facilitative effect (40% ∆Syn) using 10 µM CBD (**Supplementary Figure 7A**), which by itself did not affect cell viability (**Supplementary Figure 7B**), when co-applied with 0.1 µM doxorubicin (**Figure 6B**). A similar facilitative effect was achieved with 2-APB when co-applied with 2 µM doxorubicin (see **Figure 5**). Interestingly, higher concentrations of doxorubicin together with 10 µM CBD did not significantly change this facilitative effect (**Supplementary Figure 7A**).

### Co-Application of Doxorubicin With CBD Inhibits the Formation of BNL1 ME Cell Colonies

**Figure 7:**
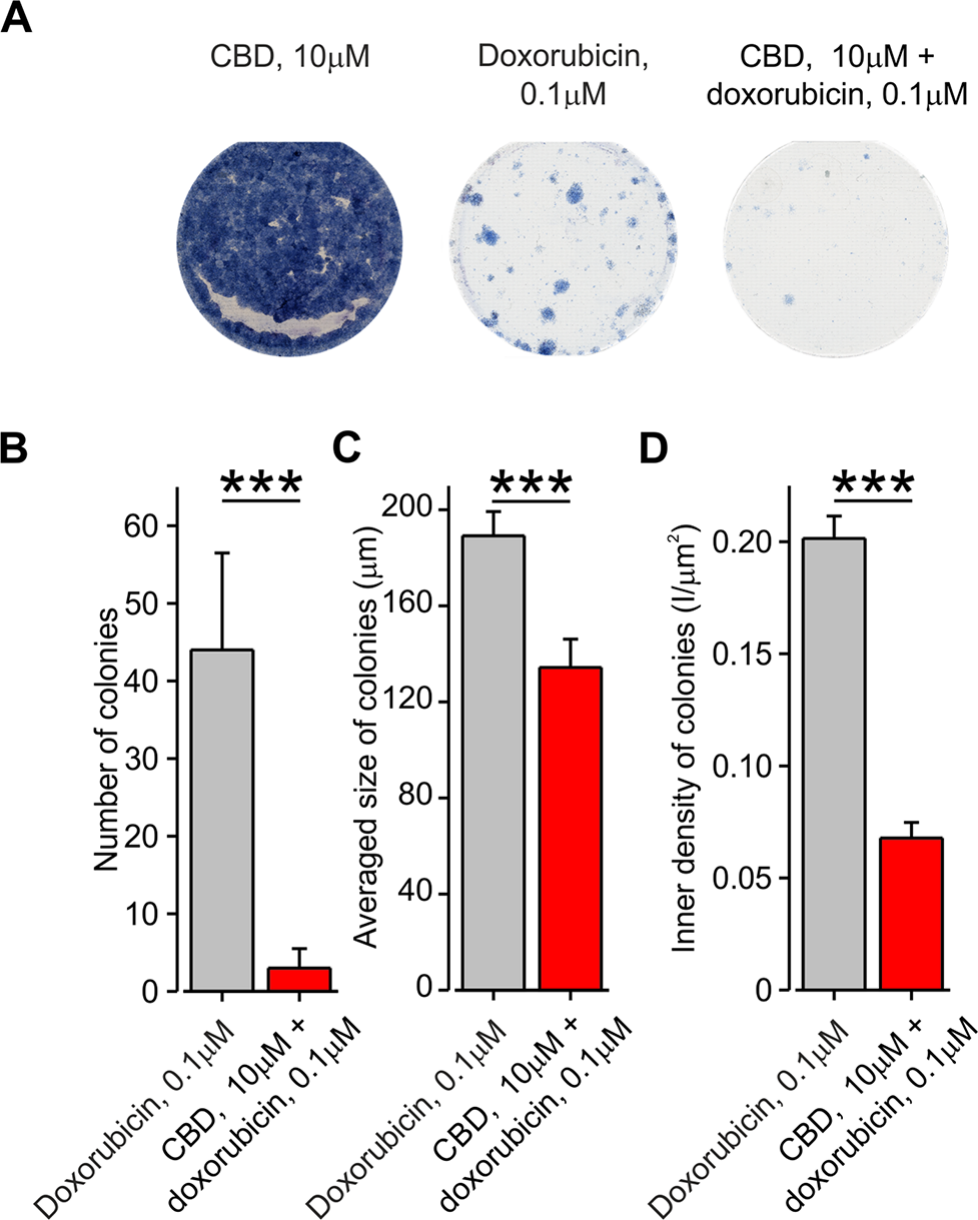
Co-application of doxorubicin with CBD, but not alone, inhibits the formation of BNL1 ME cell colonies. **(A)** A collage image of BNL1 ME cell colonies 24 h after treatment with 10 μM CBD alone (*left*), 0.1 μM doxorubicin (*middle*), and 10 μM CBD with 0.1 μM doxorubicin (*right*). **(B**–**D)** Bar graphs plotting the number of colonies **(B)**, averaged size of colonies **(C)**, and inner density of colonies **(D)** treated with either 0.1 μM doxorubicin (*gray*) or 10 μM CBD with 0.1 μM doxorubicin (*red*). ***—p < 0.001, Student’s *t-test*, n = 6 colonies for each treatment.

A decrease in the number of viable cells measured using cell survival assay may be attributed to a decrease in cell proliferation, an increase in cell death, or both. We therefore examined if facilitated entry of doxorubicin affects cell proliferation, using the clonogenic cell survival method (*see Methods*). We analyzed the colony formation of BNL1 ME cells treated with DMSO, 0.1 µM 2-APB alone, 10 µM CBD alone, and doxorubicin alone, at two different sub-effective doses (0.1 and 2 µM), which were used in the cell viability experiments in combination with either CBD or 2-APB, respectively. The number of colonies that developed 10–15 days post-treatment was quantified. The size of the individual colonies and inner density of each colony were analyzed as well. Treatment with DMSO (*data not shown*) as well as CBD led to similar high confluences of the colonies such that it was impossible to determine specific parameters describing the properties of the single colony (**Figure 7A**, *left*). Treatment with 2 µM doxorubicin alone, which we previously used together with 2-APB in the cell viability assay, completely prevented colony formation (*data not shown*). Therefore, we did not examine the effect of 2 µM doxorubicin co-applied with 2-APB and analyzed only the colonies treated with doxorubicin and those treated with a combination of CBD and doxorubicin. Treatment with 0.1 µM doxorubicin alone substantially reduced the number of colonies, the average size of the colonies, and the inner density of the colonies (**Figures 7A**, *middle*, and **B–D**). The co-application of 0.1 µM doxorubicin and 10 µM CBD, which by itself did not affect colony formation (**Figure 7A**, *left*), enhanced the effect of doxorubicin, reducing by about 20-fold the number of colonies and significantly decreasing the size and the inner density of the colonies (**Figures 7A**, *right*, **B–D**). These results suggest that, following co-application of doxorubicin and CBD, fewer cells propagate from a single surviving cell in a given time, implying that this approach reduces cell proliferation, possibly affecting also doxorubicin-resistant cell population.

Collectively, our results show that activation of TRPV2 channels provides a pathway for facilitated entry of doxorubicin into BNL1 ME cells. Our data suggest that doxorubicin, in doses that do not affect cell viability, when co-applied with TRPV2 activators, significantly reduces viability and proliferation of cancer cells.

## Discussion

The ultimate goal of developing an anticancer drug is to target cancer cells in a selective and specific manner while sparing healthy cells. To achieve this goal, some strategies are tuned to develop new drugs targeting cancer cell-specific molecular machinery. Other strategies aim at achieving specificity by using nanomedicines polymeric drug carriers such as liposomes and nanoparticles, restricting the delivery of non-specific chemotherapeutics primarily to the tumor tissues (Gabizon et al., 2014). In this study, we introduced a method of targeted delivery of cytotoxic compounds into cells harboring large-pore cation non-selective channels such as the TRPV2 channel. TRPV2 is a member of the TRP channel family. TRP channels were shown to be overexpressed by many tumor cells, playing critical roles in tumorigenesis, tumor vascularization, and the ability of the tumor cell to proliferate and migrate (Prevarskaya et al., 2007; Santoni and Farfariello, 2011; Fiorio Pla and Gkika, 2013; Chen et al., 2014). Here, we exploited these channels as cell-specific “natural” drug delivery systems for targeted application of charged molecules which are cytotoxic or antiproliferative, when inside the cells, but relatively non-toxic when outside the cells, to cancer cells, minimizing unwanted effects on other, non-TRP expressing cells.

Doxorubicin and other anthracyclines are widely used in chemotherapy, due to their efficacy in treating a broad spectrum of cancer types, such as sarcomas, carcinomas, and hematological cancers. However, their use is limited, particularly by their cardiac toxicity (Swain et al., 2003). Doxorubicin causes cumulative and mostly irreversible damage to the cardiac muscle (Ewer et al., 2004), restricting the repeated use of this drug. In addition, doxorubicin is also a prominent myelosuppressive agent. Therefore, the concentration of doxorubicin used must be strictly limited to avoid those and other doxorubicin’ side effects.

However, the weak base chemical properties of doxorubicin will reduce the rate of drug uptake, particularly in the acidic environment of cancer cells, in which doxorubicin will persist mostly in the charged form. This property of doxorubicin substantially impedes doxorubicin permeation across the membrane, such that its concentration may be around three-fold higher outside of the cell than on the inside, as suggested by some studies (Webb et al., 2011). Here, we utilized the usually ineffective charged fraction of doxorubicin and shuttled it into cancer cells *via* cation permeable TRPV2 channels, allowing us to achieve the desired effect using substantially lower doses of doxorubicin.

We and others have previously demonstrated that the pore of TRPV1 and TRPA1 channels, which are predominantly expressed by pain- and itch-related peripheral neurons, are large enough to allow passage of QX-314, a charged derivative of lidocaine that is ineffective when applied extracellularly but blocks sodium channels and consequently neuronal excitability when inside of cells (Binshtok et al., 2007; Puopolo et al., 2013; Roberson et al., 2013). By showing QX-314-mediated decrease of TRPV1-induced inward current, it was previously suggested that that activation of TRPV1 channels provided a pathway for entry of QX-314 into pain-related neurons, with QX-314 acting as a permeant blocker (Puopolo et al., 2013). Co-application of TRPV1 or TRPA1 activators enabled specific inhibition of pain and itch transmission without affecting other sensory and motor neurons (Binshtok et al., 2007; Binshtok et al., 2009b; Binshtok et al., 2009a; Roberson et al., 2013). Here, we demonstrated that the application of doxorubicin inhibited TRPV2-induced Ca^2+^ influx, which implies that doxorubicin permeates into BNL1 ME cells *via* the pore of the TRPV2 channels.

TRPV2 is overexpressed by a variety of cancer cells and plays a functional role in hepatocellular carcinoma, prostate cancer, bladder cancer, and glioblastoma development (Liu et al., 2010; Monet et al., 2010; Nabissi et al., 2013). It also is one of the factors of dystrophic cardiomyopathy (Iwata et al., 2003; Lorin et al., 2015). The latter implies that, if heart cells express functional TRPV2 channels on their membrane, the activation of TRPV2 may lead to entry of doxorubicin into heart cells and thus enhance doxorubicin-based cardiotoxicity. We, however, demonstrated that, although heart cells express a high level of TRPV2 mRNA, the expression of the TRPV2 protein in heart cells was barely detectable. Importantly, we also did not detect the expression of TRPV2 protein in liver cells.

Several explanations can be provided for the difference in expression of TRPV2 at the mRNA and protein levels. Accumulating evidence shows a weak correlation between mRNA and protein levels. Different post-transcriptional regulatory mechanisms involving cis-acting and trans-acting mechanisms generate different systems that enhance or repress the synthesis of proteins from a certain copy number of mRNA molecules (Maier et al., 2009). Moreover, mRNA levels do not always correlate with the relative expression of TRP channels at the protein level or in their functional state (Vandewauw et al., 2013). Therefore, while mRNA expression values are used in the diagnosis and classification of cancers, the abundance of proteins and their interactions are far more critical in determining cellular functionality (Greenbaum et al., 2003). However, it is noteworthy that, in order to retain normalization of the liver and heart cells, we used here normal liver and heart tissue and compared the TRPV2 expression between cell line (hepatocellular carcinoma) and these tissues. Such a comparison between cell lines and cultures tissues can by itself cause discrepancies.

To activate TRPV2 channels, we used the well-known activators of TRPV2-2-APB (Hu et al., 2004; Juvin et al., 2007) and CBD (Qin et al., 2008). We showed that the application of 2-APB onto BNL1 ME cells leads to Ca^2+^ influx in the presence of thapsigargin only when Ca^2+^ is present in the external solution. This rules out the possibility that 2-APB leads to an increase of intracellular Ca^2+^ *via* intracellular mechanisms. Notably, 2-APB activates mouse and rat TRPV2 channels with different potencies, while the human TRPV2 channel is insensitive to 2-APB when expressed in HEK293 (Neeper et al., 2007). To emphasize on the translational aspect of our findings, we have also examined other activators of TRPV2 and demonstrated that CBD-mediated activation of TRPV2 allows facilitated entry of doxorubicin into BNL1 ME cells. However, neither 2-APB nor CBD are selective TRPV2 agonists, activating other large pore channels (Bisogno et al., 2001; Hu et al., 2004; DeHaven et al., 2008). The similar effects shown here by two separate TRPV2 agonists suggest TRPV2 involvement in facilitated entry of doxorubicin into BNL1 ME cells. At this stage, we cannot exclude other possible mechanisms for doxorubicin penetration.

We show that the effect of 24-h co-treatment of doxorubicin and 2-APB on cell viability led to a decrease of about 70% in the number of living cells, while application of each of the drugs separately did not produce any significant cell death, suggesting a facilitative effect of 2-APB (see **Figure 5D**). However, considering that prolonged treatment with 2-APB or doxorubicin did produce a toxic effect (as shown in **Supplementary Figure 5**), an alternative explanation could be that the 24-h application of 2-APB and/or doxorubicin did not kill the cells but stressed them in a way which was not detected by the live essay. In this case, the cumulative effect of co-application might have led to cell death. Our data showing that the substantial facilitative effect, which was achieved using a relatively less toxic activator, CBD, suggests that the latter explanation is less likely and implies that co-application of TRPV2 activators with doxorubicin leads to facilitative entry of doxorubicin and thus increased BNL1 ME cell death.

The effect of 2-APB and doxorubicin, applied alone, on the cell viability after 48 and 72 h, suggests that that the optimal effect of 2-APB-mediated facilitated entry of doxorubicin on cell vitality is achieved at 24 h after initiation of treatment. Systemic application of doxorubicin reaches maximal concentrations within 30 to 60 min and is cleared very quickly from the organism (Gabizon et al., 2003). Thus, the 24-h facilitative window could be achieved by either prolonged infusion of doxorubicin together with the channel activators or by using slow-released pegylated liposomal doxorubicin (Tahover et al., 2015), paired with prolonged infusion of the channel agonist.

Importantly, according to the human protein atlas database (Uhlen et al., 2015), TRPV2 is highly expressed in many organs, such as endocrine tissues, kidneys, and skin, but not in the liver cells. These data imply that, in translation from our proof-of-concept results to the clinic, local liver application of the treatment would assure a specific effect on hepatocellular carcinoma cells but not an off-target effect of normal liver cells (as they do not express TRPV2 protein), and no other tissues, by virtue of its local application.

Our data from transfected HEK293T cells expressing TRPV2 demonstrate that CBD-mediated activation of TRPV2 channels leads to doxorubicin entry into TRPV2 expressing HEK293T cells *via* the pore of TRPV2 channels, emphasizing that TRPV2 could be utilized as natural drug delivery system. Importantly, since CBD is widely used in the clinic (Kogan and Mechoulam, 2007), our results showing CBD-mediated facilitated entry of doxorubicin opens up the possibility of clinical utilization of the platform we describe here.

The observed effect of CBD together with doxorubicin on BNL1 ME cell viability was significantly stronger than the effect of 2-APB together with doxorubicin. We, therefore, assumed that CBD might have an additional facilitative effect on tumor cell death. We ruled out an effect of CBD alone on cell viability. Our next assumption was that, in addition to the facilitation of doxorubicin entry, CBD might also affect doxorubicin removal from cells. Indeed, CBD was demonstrated to inhibit the P-gp ATPase, which affects the removal of doxorubicin from the cells (Zhu et al., 2006). Using the well-described P-gp ATPase antagonist verapamil (Cornwell et al., 1987), we show that (1) BNL1 ME cells, similarly to many tumor cells (Kartner et al., 1983), express functional P-gp ATPase, and (2) blockade of P-gp ATPase by verapamil leads to increase in doxorubicin concentration. These results render P-gp ATPase as a possible target for CBD inhibition, thus underlying the substantial effects of previously ineffective 0.1 µM doxorubicin on cell viability. Moreover, our data showing that the application of CBD together with doxorubicin leads to an increase in doxorubicin concentration which was significantly higher than when doxorubicin was co-applied with verapamil emphasizes the importance of CBD-induced, TRPV2-mediated facilitated entry of doxorubicin into BNL1 ME cells.

The effect of TRPV2-mediated entry of doxorubicin on colony survival, in addition to its effects on cell viability, may also be significant in terms of drug resistance to doxorubicin (Broxterman et al., 2009). It is possible that the colonies which survived the application of doxorubicin alone were generated from cells with resistance to doxorubicin. Importantly, these colonies were annulled when doxorubicin was co-applied with CBD, implying that this combined platform could potentially be beneficial against doxorubicin drug resistance. Since P-gp transporter expression was correlated with drug resistance in cancer cells (Kartner et al., 1983), CBD-mediated inhibition of P-gp ATPase could underlie this effect of CBD and doxorubicin on inhibition of cell colonies in addition to TRPV2-mediated entry of doxorubicin.

In summary, we have demonstrated here a platform for facilitated entry of doxorubicin into cancer cells. Such facilitated entry may allow the use of lower doses of the cytotoxic agent minimizing the off-target toxic effects and therefore may allow an improved therapeutic index. The methodology proposed here is based on the biological properties of specific cells harboring large cationic channels. These large-pore cationic channels are expressed at high levels by cancer cells provide a “natural” drug delivery system. This drug delivery system, when activated, could be used to introduce cytotoxic drugs selectively into cancer cells. Thus, our approach suggests a novel method for the delivery of a variety of drugs that are (1) selective to the target cells and (2) will not require complex manipulations of cells or drugs. Another important aspect of the strategy is that it promises to open up a large new chemical space of potential anti-cancer agents (cytotoxic, antiproliferative, as well as anticancer peptides and nucleic acid medicines), i.e., cationic molecules that are poorly cell-permeant, to be effectively introduced by permeation through large cationic channels. The results of our work may provide a basis for the development of novel tools to modulate intracellular signal transduction and metabolic pathways and thereby to treat cancer with significantly fewer side effects and higher efficacy. The data we show, in conjunction with the differential expression profile of large pore cation non-selective channels, as seen in the human protein atlas database (Uhlen et al., 2015), and data related to differential expression of these channels in cancer cells (*de novo*, expression, upregulation, or downregulation; *see for example* Shapovalov et al., 2016), would suggest that the translation of our platform is not trivial. It implies the need for careful profiling when considering this strategy for different cancers.

## Data Availability Statement

The raw data supporting the conclusions of this manuscript will be made available by the authors, without undue reservation, to any qualified researcher.

## Author Contributions

Conceptualization, AB; Methodology, AL, YS, RK, AG, AH, and AB; Investigation, HN-R, AS, SL, MM, BK, DS; Writing - Original Draft, HN-R, AL, YS, RK, AG, AH, and AB; Funding Acquisition, AB; Supervision, AB.

## Funding

Support is gratefully acknowledged from the Deutsch-Israelische Projectkooperation program of the Deutsche Forschungsgemeinschaft (DIP) grant agreement BI 1665/1-1ZI1172/12-1 (HN-R, BK, SL, and AB); the Israeli Science Foundation - grant agreement 1470/17 (HN-R, BK, SL and AB); Rosetrees Foundation A1777 and M139 (AB) and the Marie Curie International Reintegration Grant and Rosetrees Trust (AB).

## Conflict of Interest

The authors declare that the research was conducted in the absence of any commercial or financial relationships that could be construed as a potential conflict of interest.

## References

[B1] Bagnyukova T. V. Serebriiskii I. G. Zhou Y. Hopper-Borge E. A. Golemis E. A. Astsaturov I. (2010). Chemotherapy and signaling: how can targeted therapies supercharge cytotoxic agents? Cancer Biol. Ther. 10, 839–853. 10.4161/cbt.10.9.13738 20935499 PMC3012138

[B2] Bean B. P. Binshtok A. M. Woolf C. J. (2007). Harvard Office of Technology Development: targeting anti-cancer drugs intracellularly *via* TRP channels. http://www.techtransfer.harvard.edu/technologies/tech.php?​case=3059.

[B3] Binshtok A. M. Bean B. P. Woolf C. J. (2007). Inhibition of nociceptors by TRPV1-mediated entry of impermeant sodium channel blockers. Nature 449, 607–610. 10.1038/nature06191 17914397

[B4] Binshtok A. M. Roberson D. P. Bean B. P. Woolf C. J. (2009a). . Activation of TRPA1 as well as TRPV1 channels by lidocaine allows entry of QX-314 into nociceptors to produce a pain selective sodium channel block. Chicago, IL: Society for Neuroscience Meeting.

[B5] Binshtok A. M. Gerner P. Oh S. B. Puopolo M. Suzuki S. Roberson D. P. (2009b). Coapplication of lidocaine and the permanently charged sodium channel blocker QX-314 produces a long-lasting nociceptive blockade in rodents. Anesthesiology 111, 127–137. 10.1097/ALN.0b013e3181a915e7 19512868 PMC2761747

[B6] Bisogno T. Hanus L. De Petrocellis L. Tchilibon S. Ponde D. E. Brandi I. (2001). Molecular targets for cannabidiol and its synthetic analogues: effect on vanilloid VR1 receptors and on the cellular uptake and enzymatic hydrolysis of anandamide. Br. J. Pharmacol. 134, 845–852. 10.1038/sj.bjp.0704327 11606325 PMC1573017

[B7] Broxterman H. J. Gotink K. J. Verheul H. M. (2009). Understanding the causes of multidrug resistance in cancer: a comparison of doxorubicin and sunitinib. Drug Resist Update 12, 114–126. 10.1016/j.drup.2009.07.001 19648052

[B8] Bruix J. Sherman M. (2011). Management of hepatocellular carcinoma: an update. Hepatology 53, 1020–1022. 10.1002/hep.24199 21374666 PMC3084991

[B9] Caprodossi S. Lucciarini R. Amantini C. Nabissi M. Canesin G. Ballarini P. (2008). Transient receptor potential vanilloid type 2 (TRPV2) expression in normal urothelium and in urothelial carcinoma of human bladder: correlation with the pathologic stage. Eur. Urol. 54, 612–620. 10.1016/j.eururo.2007.10.016 17977643

[B10] Chatterjee K. Zhang J. Honbo N. Karliner J. S. (2010). Doxorubicin cardiomyopathy. Cardiology 115 (2), 155–162. 10.1159/000265166 20016174 PMC2848530

[B11] Chen J. Luan Y. Yu R. Zhang Z. Zhang J. Wang W. (2014). Transient receptor potential (TRP) channels, promising potential diagnostic and therapeutic tools for cancer. Biosci. Trends 8, 1–10. 10.5582/bst.8.1 24647107

[B12] Cornwell M. M. Pastan I. Gottesman M. M. (1987). Certain calcium channel blockers bind specifically to multidrug-resistant human KB carcinoma membrane vesicles and inhibit drug binding to P-glycoprotein. J. Biol. Chem. 262, 2166–2170. 2434476

[B13] DeHaven W. I. Smyth J. T. Boyles R. R. Bird G. S. Putney J. W. Jr. (2008). Complex actions of 2-aminoethyldiphenyl borate on store-operated calcium entry. J. Biol. Chem. 283, 19265–19273. 10.1074/jbc.M801535200 18487204 PMC2443677

[B14] Dobbelstein M. Moll U. (2014). Targeting tumour-supportive cellular machineries in anticancer drug development. Nat. Rev. Drug Discov. 13, 179–196. 10.1038/nrd4201 24577400

[B15] Eubler K. Herrmann C. Tiefenbacher A. Kohn F. M. Schwarzer J. U. Kunz L. (2018). Ca(2+) Signaling and IL-8 secretion in human testicular peritubular cells involve the cation channel TRPV2. Int. J. Mol. Sci. 19 (9), 2829. 10.3390/ijms19092829 PMC6165404 30235802

[B16] Ewer M. S. Martin F. J. Henderson C. Shapiro C. L. Benjamin R. S. Gabizon A. A. (2004). Cardiac safety of liposomal anthracyclines. Semin. Oncol. 31, 161–181. 10.1053/j.seminoncol.2004.08.006 15717742

[B17] Ferry D. R. (1998). Testing the role of P-glycoprotein expression in clinical trials: applying pharmacological principles and best methods for detection together with good clinical trials methodology. Int. J. Clin. Pharmacol. Ther. 36, 29–40. 9476146

[B18] Fiorio Pla A. Gkika D. (2013). Emerging role of TRP channels in cell migration: from tumor vascularization to metastasis. Front. Physiol. 4, 311. 10.3389/fphys.2013.00311 24204345 PMC3817680

[B19] Gabizon A. Shmeeda H. Barenholz Y. (2003). Pharmacokinetics of pegylated liposomal doxorubicin: review of animal and human studies. Clin. Pharmacokinet. 42, 419–436. 10.2165/00003088-200342050-00002 12739982

[B20] Gabizon A. Bradbury M. Prabhakar U. Zamboni W. Libutti S. Grodzinski P. (2014). Cancer nanomedicines: closing the translational gap. Lancet 384, 2175–2176. 10.1016/S0140-6736(14)61457-4 25625382 PMC6615547

[B21] Gallagher F. A. Kettunen M. I. Day S. E. Hu D. E. Ardenkjaer-Larsen J. H. Zandt R. (2008). Magnetic resonance imaging of pH *in vivo* using hyperpolarized 13C-labelled bicarbonate. Nature 453, 940–943. 10.1038/nature07017 18509335

[B22] Giavazzi R. Kartner N. Hart I. R. (1984). Expression of cell surface P-glycoprotein by an adriamycin-resistant murine fibrosarcoma. Cancer Chemother. Pharmacol. 13, 145–147. 10.1007/BF00257134 6147205

[B23] Greenbaum D. Colangelo C. Williams K. Gerstein M. (2003). Comparing protein abundance and mRNA expression levels on a genomic scale. Genome Biol. 4, 117. 10.1186/gb-2003-4-9-117 12952525 PMC193646

[B24] Hollo Z. Homolya L. Davis C. W. Sarkadi B. (1994). Calcein accumulation as a fluorometric functional assay of the multidrug transporter. Biochim. Biophys. Acta 1191, 384–388. 10.1016/0005-2736(94)90190-2 7909692

[B25] Hu H. Z. Gu Q. Wang C. Colton C. K. Tang J. Kinoshita-Kawada M. (2004). 2-aminoethoxydiphenyl borate is a common activator of TRPV1, TRPV2, and TRPV3. J. Biol. Chem. 279, 35741–35748. 10.1074/jbc.M404164200 15194687

[B26] Iwata Y. Katanosaka Y. Arai Y. Komamura K. Miyatake K. Shigekawa M. (2003). A novel mechanism of myocyte degeneration involving the Ca2+-permeable growth factor-regulated channel. J. Cell Biol. 161, 957–967. 10.1083/jcb.200301101 12796481 PMC2172975

[B27] Juvin V. Penna A. Chemin J. Lin Y. L. Rassendren F. A. (2007). Pharmacological characterization and molecular determinants of the activation of transient receptor potential V2 channel orthologs by 2-aminoethoxydiphenyl borate. Mol. Pharmacol. 72, 1258–1268. 10.1124/mol.107.037044 17673572

[B28] Karni R. Jove R. Levitzki A. (1999). Inhibition of pp60c-Src reduces Bcl-XL expression and reverses the transformed phenotype of cells overexpressing EGF and HER-2 receptors. Oncogene 18, 4654–4662. 10.1038/sj.onc.1202835 10467412

[B29] Karni R. de Stanchina E. Lowe S. W. Sinha R. Mu D. Krainer A. R. (2007). The gene encoding the splicing factor SF2/ASF is a proto-oncogene. Nat. Struct. Mol. Biol. 14, 185–193. 10.1038/nsmb1209 17310252 PMC4595851

[B30] Kartner N. Riordan J. R. Ling V. (1983). Cell surface P-glycoprotein associated with multidrug resistance in mammalian cell lines. Science 221, 1285–1288. 10.1126/science.6137059 6137059

[B31] Kogan N. M. Mechoulam R. (2007). Cannabinoids in health and disease. Dialogues Clin. Neurosci. 9, 413–430. 18286801 10.31887/DCNS.2007.9.4/nkogan PMC3202504

[B32] Kuriyama S. Masui K. Kikukawa M. Sakamoto T. Nakatani T. Nagao S. (1999). Complete cure of established murine hepatocellular carcinoma is achievable by repeated injections of retroviruses carrying the herpes simplex virus thymidine kinase gene. Gene Ther. 6, 525–533. 10.1038/sj.gt.3300869 10476212

[B33] Lal S. Mahajan A. Chen W. N. Chowbay B. (2010). Pharmacogenetics of target genes across doxorubicin disposition pathway: a review. Curr. Drug Metab. 11, 115–128. 10.2174/138920010791110890 20302569

[B34] Liberati S. Morelli M. B. Amantini C. Santoni M. Nabissi M. Cardinali C. (2014). Advances in transient receptor potential vanilloid-2 channel expression and function in tumor growth and progression. Curr. Protein Pept. Sci. 15, 732–737. 10.2174/1389203715666140704115913 25001513

[B35] Liu G. Xie C. Sun F. Xu X. Yang Y. Zhang T. (2010). Clinical significance of transient receptor potential vanilloid 2 expression in human hepatocellular carcinoma. Cancer Gene. Cytogenet. 197, 54–59. 10.1016/j.cancergencyto.2009.08.007 20113837

[B36] Lorin C. Vogeli I. Niggli E. (2015). Dystrophic cardiomyopathy: role of TRPV2 channels in stretch-induced cell damage. Cardiovasc Res. 106, 153–162. 10.1093/cvr/cvv021 25616416

[B37] Maier T. Guell M. Serrano L. (2009). Correlation of mRNA and protein in complex biological samples. FEBS Lett. 583, 3966–3973. 10.1016/j.febslet.2009.10.036 19850042

[B38] Maruyama T. Kanaji T. Nakade S. Kanno T. Mikoshiba K. (1997). 2APB, 2-aminoethoxydiphenyl borate, a membrane-penetrable modulator of Ins(1,4,5)P3-induced Ca2+ release. J. Biochem. 122, 498–505. 10.1093/oxfordjournals.jbchem.a021780 9348075

[B39] Mizuno H. Suzuki Y. Watanabe M. Sokabe T. Yamamoto T. Hattori R. (2014). Potential role of transient receptor potential (TRP) channels in bladder cancer cells. J. Physiol. Sci. 64, 305–314. 10.1007/s12576-014-0319-6 24849279 PMC10717035

[B40] Monet M. Lehen’kyi V. Gackiere F. Firlej V. Vandenberghe M. Roudbaraki M. (2010). Role of cationic channel TRPV2 in promoting prostate cancer migration and progression to androgen resistance. Cancer Res. 70, 1225–1235. 10.1158/0008-5472.CAN-09-2205 20103638

[B41] Nabissi M. Morelli M. B. Santoni M. Santoni G. (2013). Triggering of the TRPV2 channel by cannabidiol sensitizes glioblastoma cells to cytotoxic chemotherapeutic agents. Carcinogenesis 34, 48–57. 10.1093/carcin/bgs328 23079154

[B42] Nabissi M. Morelli M. B. Amantini C. Farfariello V. Ricci-Vitiani L. Caprodossi S. (2010). TRPV2 channel negatively controls glioma cell proliferation and resistance to Fas-induced apoptosis in ERK-dependent manner. Carcinogenesis 31, 794–803. 10.1093/carcin/bgq019 20093382

[B43] Neeper M. P. Liu Y. Hutchinson T. L. Wang Y. Flores C. M. Qin N. (2007). Activation properties of heterologously expressed mammalian TRPV2: evidence for species dependence. J. Biol. Chem. 282, 15894–15902. 10.1074/jbc.M608287200 17395593

[B44] Ogunwobi O. O. Liu C. (2011). Hepatocyte growth factor upregulation promotes carcinogenesis and epithelial-mesenchymal transition in hepatocellular carcinoma *via* Akt and COX-2 pathways. Clin. Exp Metastasis 28, 721–731. 10.1007/s10585-011-9404-x 21744257 PMC3732749

[B45] Patil Y. Shmeeda H. Amitay Y. Ohana P. Kumar S. Gabizon A. (2018). Targeting of folate-conjugated liposomes with co-entrapped drugs to prostate cancer cells *via* prostate-specific membrane antigen (PSMA). Nanomedicine 14, 1407–1416. 10.1016/j.nano.2018.04.011 29680672

[B46] Pottosin I. Delgado-Enciso I. Bonales-Alatorre E. Nieto-Pescador M. G. Moreno-Galindo E. G. Dobrovinskaya O. (2015). Mechanosensitive Ca(2)(+)-permeable channels in human leukemic cells: pharmacological and molecular evidence for TRPV2. Biochim. Biophys. Acta 1848, 51–59. 10.1016/j.bbamem.2014.09.008 25268680

[B47] Prevarskaya N. Zhang L. Barritt G. (2007). TRP channels in cancer. Biochim. Biophys. Acta 1772, 937–946. 10.1016/j.bbadis.2007.05.006 17616360

[B48] Puopolo M. Binshtok A. M. Yao G. L. Oh S. B. Woolf C. J. Bean B. P. (2013). Permeation and block of TRPV1 channels by the cationic lidocaine derivative QX-314. J. Neurophysiol. 109, 1704–1712. 10.1152/jn.00012.2013 23303863 PMC3628012

[B49] Qin N. Neeper M. P. Liu Y. Hutchinson T. L. Lubin M. L. Flores C. M. (2008). TRPV2 is activated by cannabidiol and mediates CGRP release in cultured rat dorsal root ganglion neurons. J. Neurosci. 28, 6231–6238. 10.1523/JNEUROSCI.0504-08.2008 18550765 PMC6670541

[B50] Roberson D. P. Binshtok A. M. Blasl F. Bean B. P. Woolf C. J. (2011). Targeting of sodium channel blockers into nociceptors to produce long-duration analgesia: a systematic study and review. Br. J. Pharmacol. 164, 48–58. 10.1111/j.1476-5381.2011.01391.x 21457220 PMC3171859

[B51] Roberson D. P. Gudes S. Sprague J. M. Patoski H. A. Robson V. K. Blasl F. (2013). Activity-dependent silencing reveals functionally distinct itch-generating sensory neurons. Nat. Neurosci. 16, 910–918. 10.1038/nn.3404 23685721 PMC3695070

[B52] Santoni G. Farfariello V. (2011). TRP channels and cancer: new targets for diagnosis and chemotherapy. Endocr. Metab. Immune Disord. Drug Targets 11, 54–67. 10.2174/187153011794982068 21348820

[B53] Scalori V. Alessandri M. G. Giovannini L. Bertelli A. A. Mian M. (1988). Adriamycin, aclacinomycin and thepirubicin intracardiac distribution examined by fluorescence microscopy. Chemioterapia 7, 179–183. 3168073

[B54] Shapovalov G. Ritaine A. Skryma R. Prevarskaya N. (2016). Role of TRP ion channels in cancer and tumorigenesis. Semin. Immunopathol. 38, 357–369. 10.1007/s00281-015-0525-1 26842901

[B55] Shneor D. Folberg R. Pe’er J. Honigman A. Frenkel S. (2017). Stable knockdown of CREB, HIF-1 and HIF-2 by replication-competent retroviruses abrogates the responses to hypoxia in hepatocellular carcinoma. Cancer Gene Ther. 24, 64–74. 10.1038/cgt.2016.68 27934882 PMC5339434

[B56] Singal P. K. Iliskovic N. (1998). Doxorubicin-induced cardiomyopathy. N. Engl. J. Med. 339, 900–905. 10.1056/NEJM199809243391307 9744975

[B57] Swain S. M. Whaley F. S. Ewer M. S. (2003). Congestive heart failure in patients treated with doxorubicin: a retrospective analysis of three trials. Cancer 97, 2869–2879. 10.1002/cncr.11407 12767102

[B58] Tacar O. Sriamornsak P. Dass C. R. (2013). Doxorubicin: an update on anticancer molecular action, toxicity and novel drug delivery systems. J. Pharm. Pharmacol. 65, 157–170. 10.1111/j.2042-7158.2012.01567.x 23278683

[B59] Tahover E. Patil Y. P. Gabizon A. A. (2015). Emerging delivery systems to reduce doxorubicin cardiotoxicity and improve therapeutic index: focus on liposomes. Anticancer Drugs 26, 241–258. 10.1097/CAD.0000000000000182 25415656

[B60] Tatsumi T. Takehara T. Kanto T. Kuzushita N. Ito A. Kasahara A. (1999). B7-1 (CD80)-gene transfer combined with interleukin-12 administration elicits protective and therapeutic immunity against mouse hepatocellular carcinoma. Hepatology 30, 422–429. 10.1002/hep.510300219 10421650

[B61] Thastrup O. Cullen P. J. Drobak B. K. Hanley M. R. Dawson A. P. (1990). Thapsigargin, a tumor promoter, discharges intracellular Ca2+ stores by specific inhibition of the endoplasmic reticulum Ca2(+)-ATPase. Proc. Natl. Acad. Sci. U. S. A. 87, 2466–2470. 10.1073/pnas.87.7.2466 2138778 PMC53710

[B62] Uhlén M. Fagerberg L. Hallström B.M. Lindskog C. Oksvold P. Mardinoglu A. (2015). Proteomics. Tissue-based map of the human proteome. Science 347, 1260419. 10.1126/science.1260419 25613900

[B63] Vandewauw I. Owsianik G. Voets T. (2013). Systematic and quantitative mRNA expression analysis of TRP channel genes at the single trigeminal and dorsal root ganglion level in mouse. BMC Neurosci. 14, 21. 10.1186/1471-2202-14-21 23410158 PMC3576292

[B64] Watanabe H. Murakami M. Ohba T. Ono K. Ito H. (2009). The pathological role of transient receptor potential channels in heart disease. Circ J. 73, 419–427. 10.1253/circj.CJ-08-1153 19202304

[B65] Webb B. A. Chimenti M. Jacobson M. P. Barber D. L. (2011). Dysregulated pH: a perfect storm for cancer progression. Nat. Rev. Cancer. 11 (9), 671–677. 10.1038/nrc3110 21833026

[B66] Zhou K. Zhang S. S. Yan Y. Zhao S. (2014). Overexpression of transient receptor potential vanilloid 2 is associated with poor prognosis in patients with esophageal squamous cell carcinoma. Med. Oncol. 31, 17. 10.1007/s12032-014-0017-5 24878697

[B67] Zhu H. J. Wang J, S. Markowitz J. S. Donovan J, L. Gibson B. Gefroh H. Devane C. L. (2006). Characterization of P-glycoprotein inhibition by major cannabinoids from marijuana. J. Pharmacol. Exp. Ther. 317, 850–857. 16439618 10.1124/jpet.105.098541

