# Supplementary figures and images for: 2-APB and CBD-Mediated Targeting of Charged Cytotoxic Compounds Into Tumor Cells Suggests the Involvement of TRPV2 Channels
Source: Front Pharmacol. 2019 Oct 15;10:1198. doi: 10.3389/fphar.2019.01198 (PMC6804401; doi:10.3389/fphar.2019.01198)

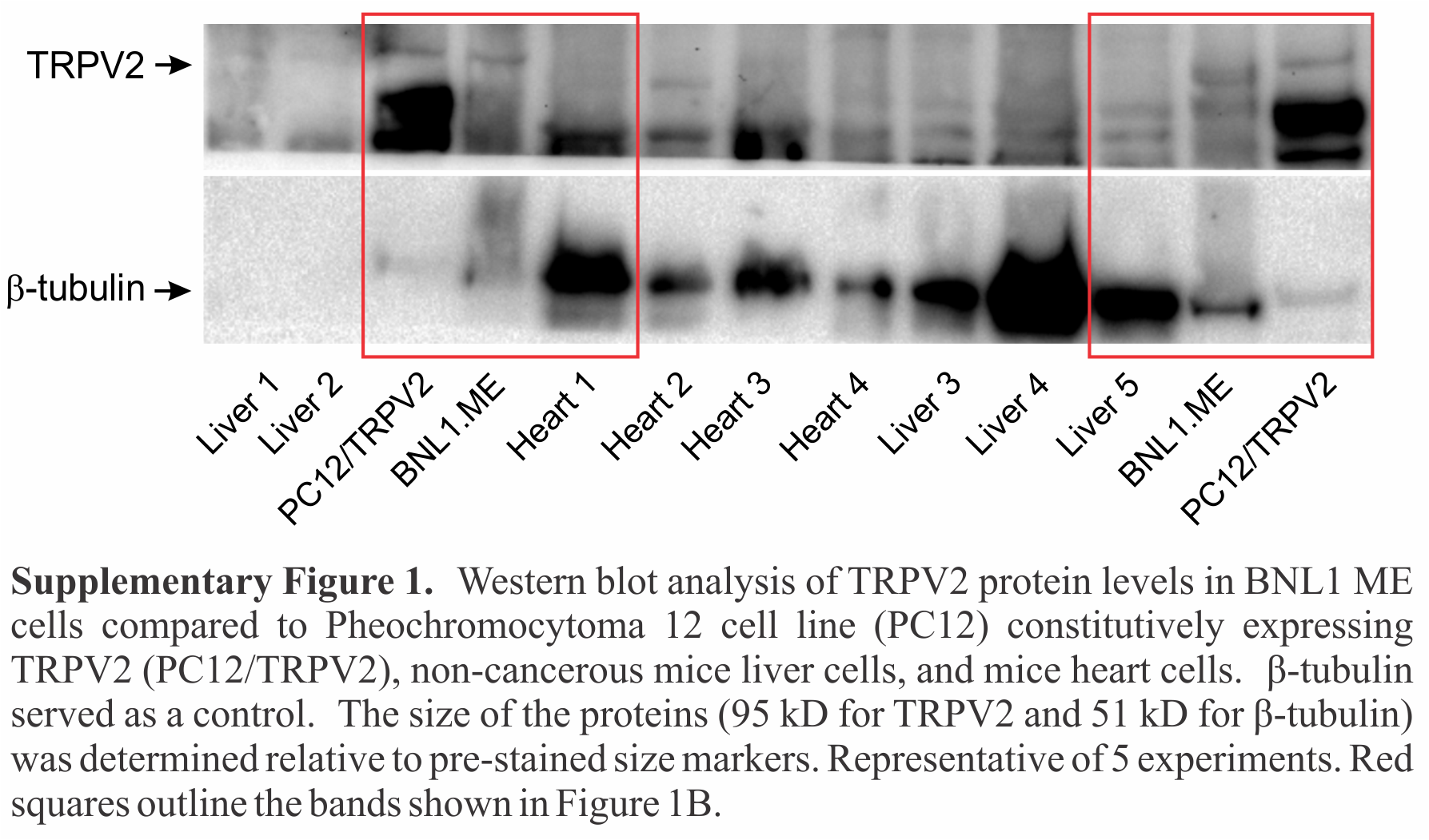

Supplement: Supplementary file 1 [file Image_1.tif]

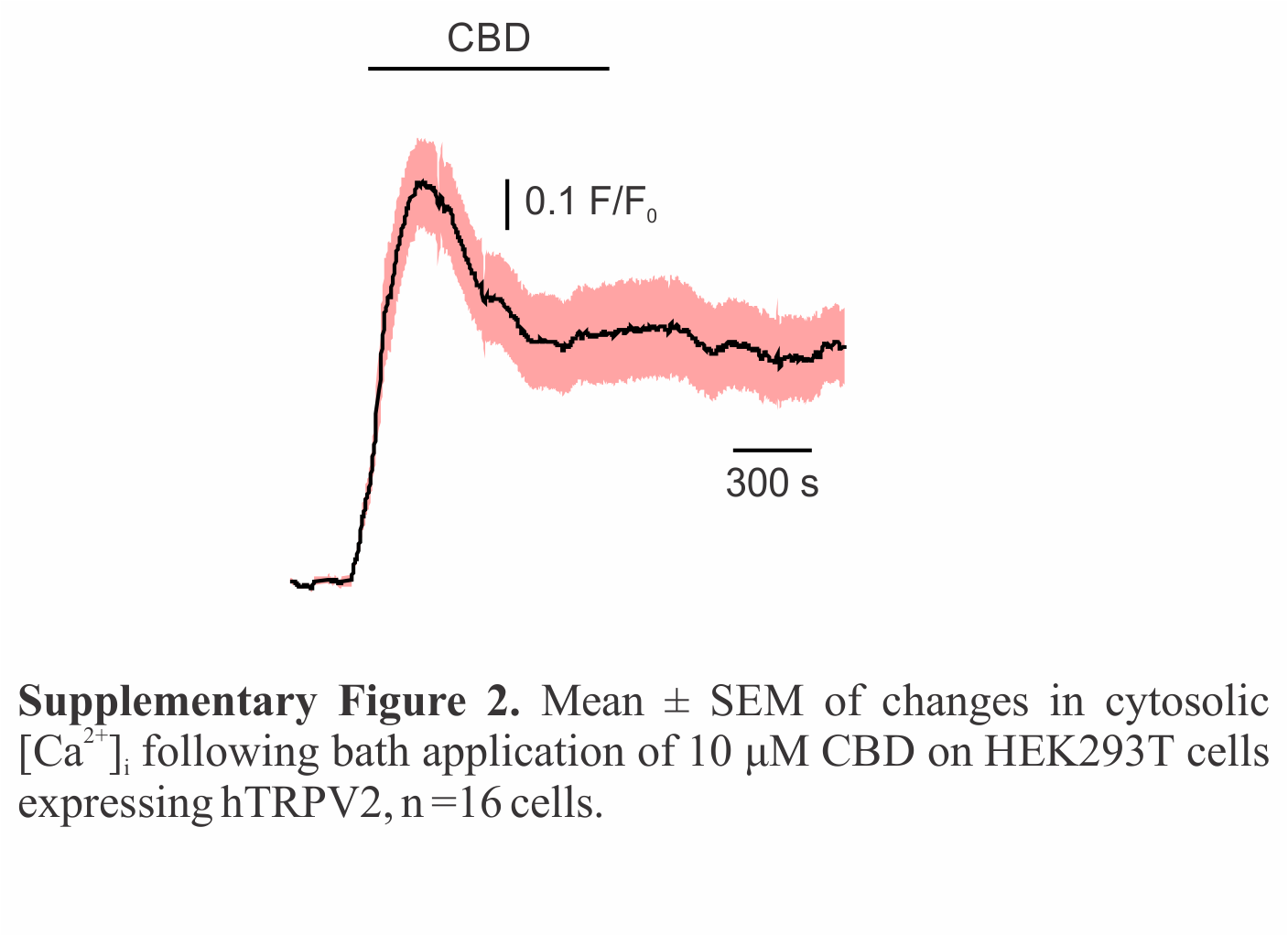

Supplement: Supplementary file 2 [file Image_2.tif]

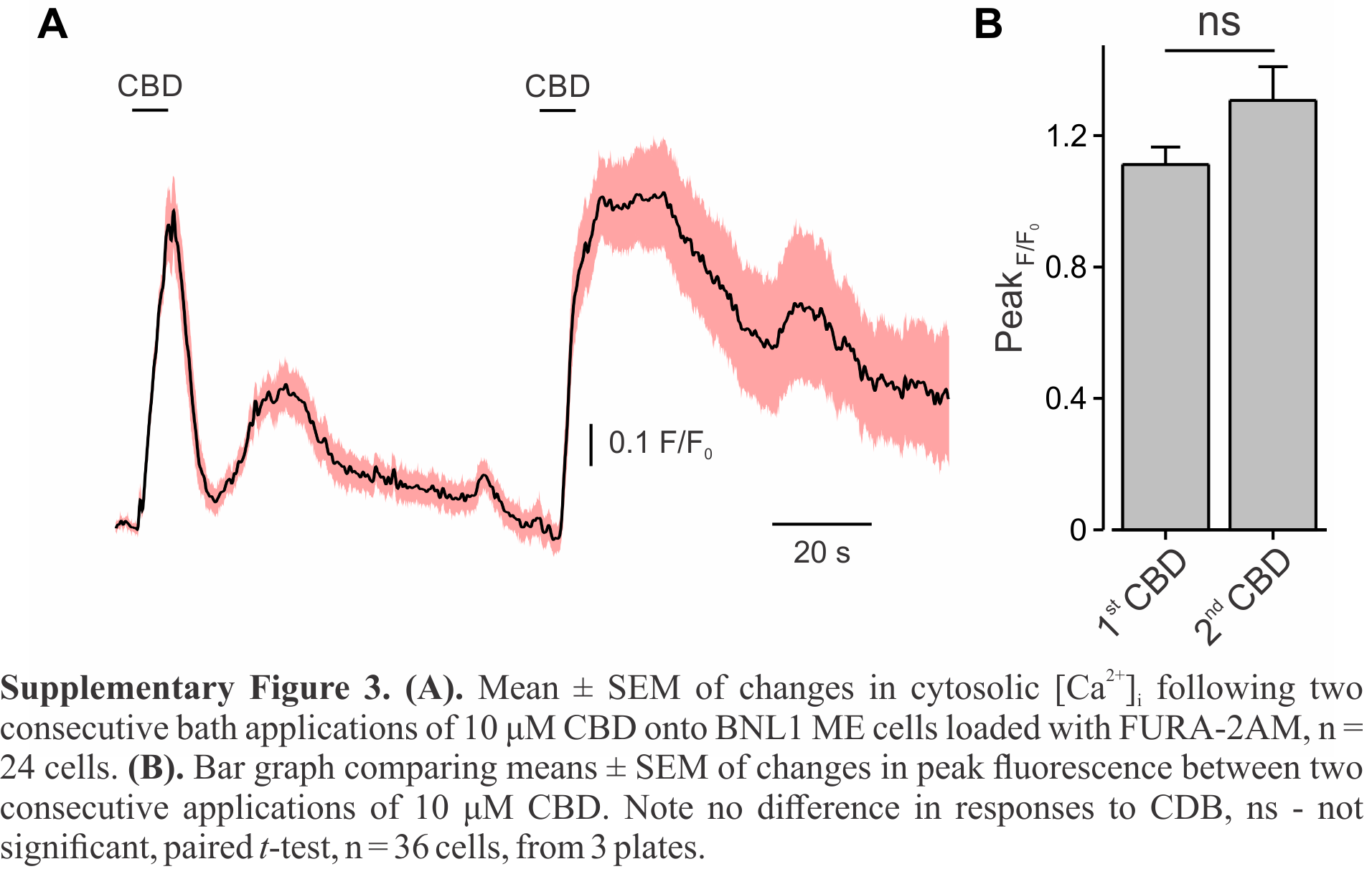

Supplement: Supplementary file 3 [file Image_3.tif]

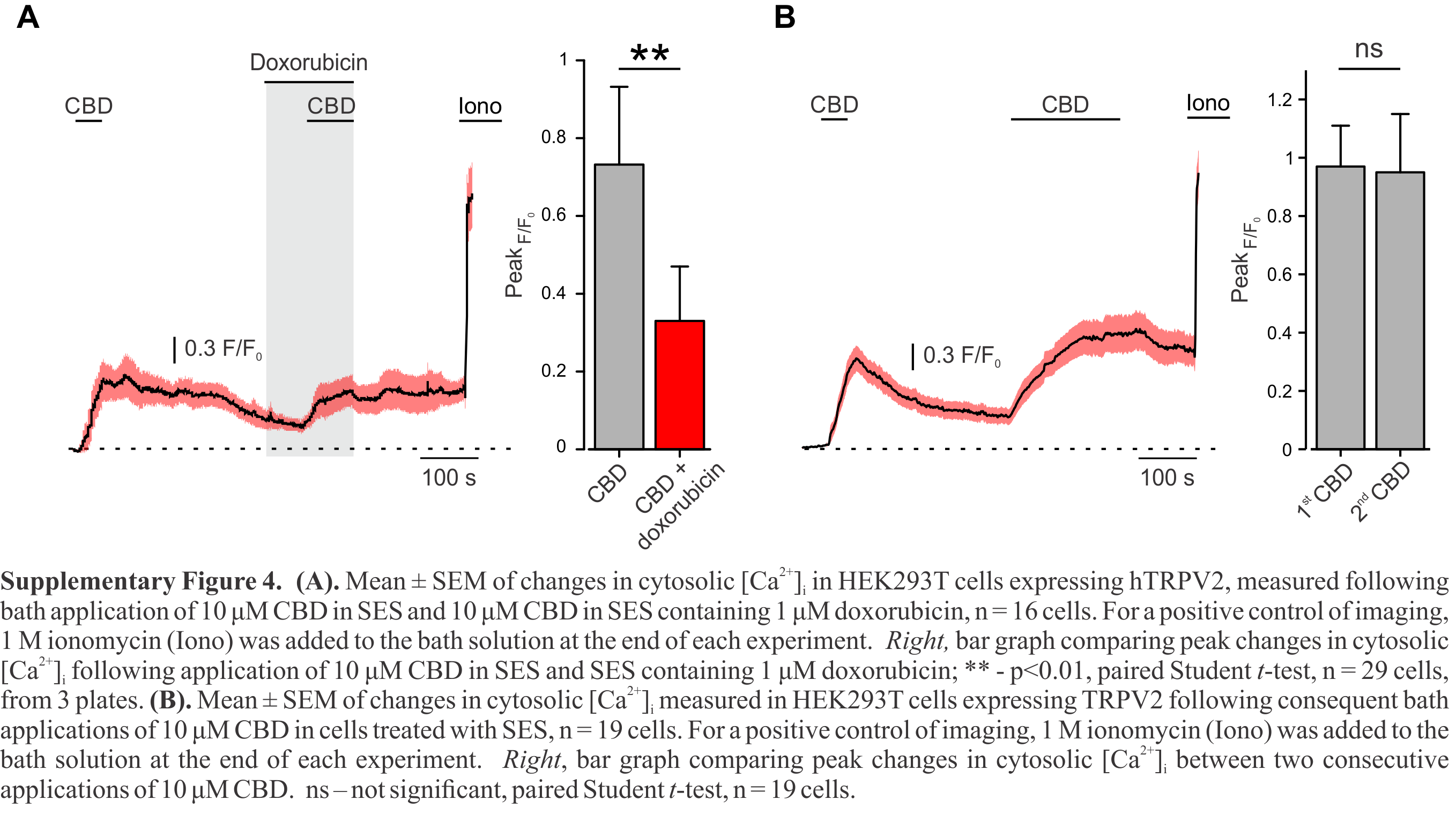

Supplement: Supplementary file 4 [file Image_4.tif]

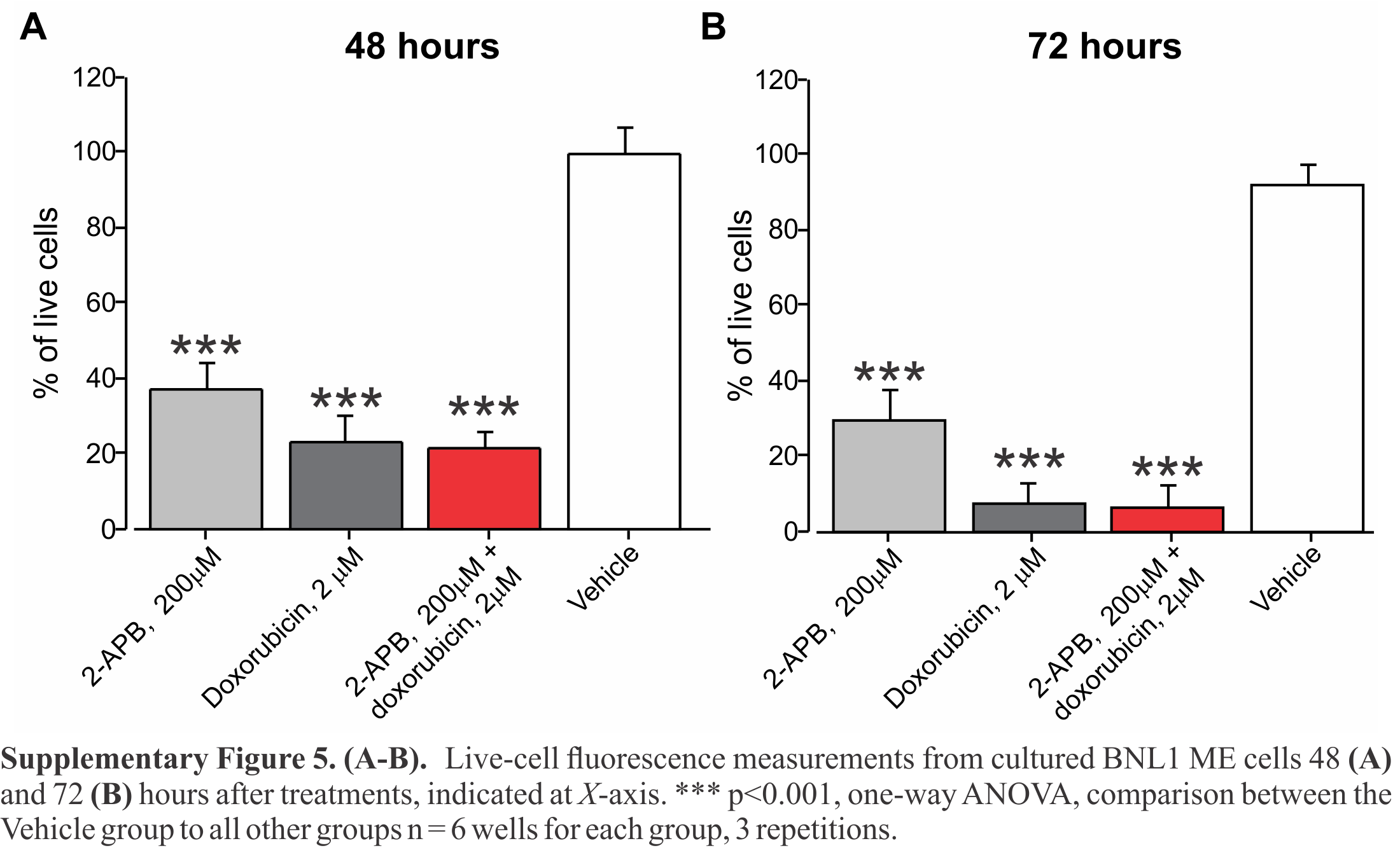

Supplement: Supplementary file 5 [file Image_5.tif]

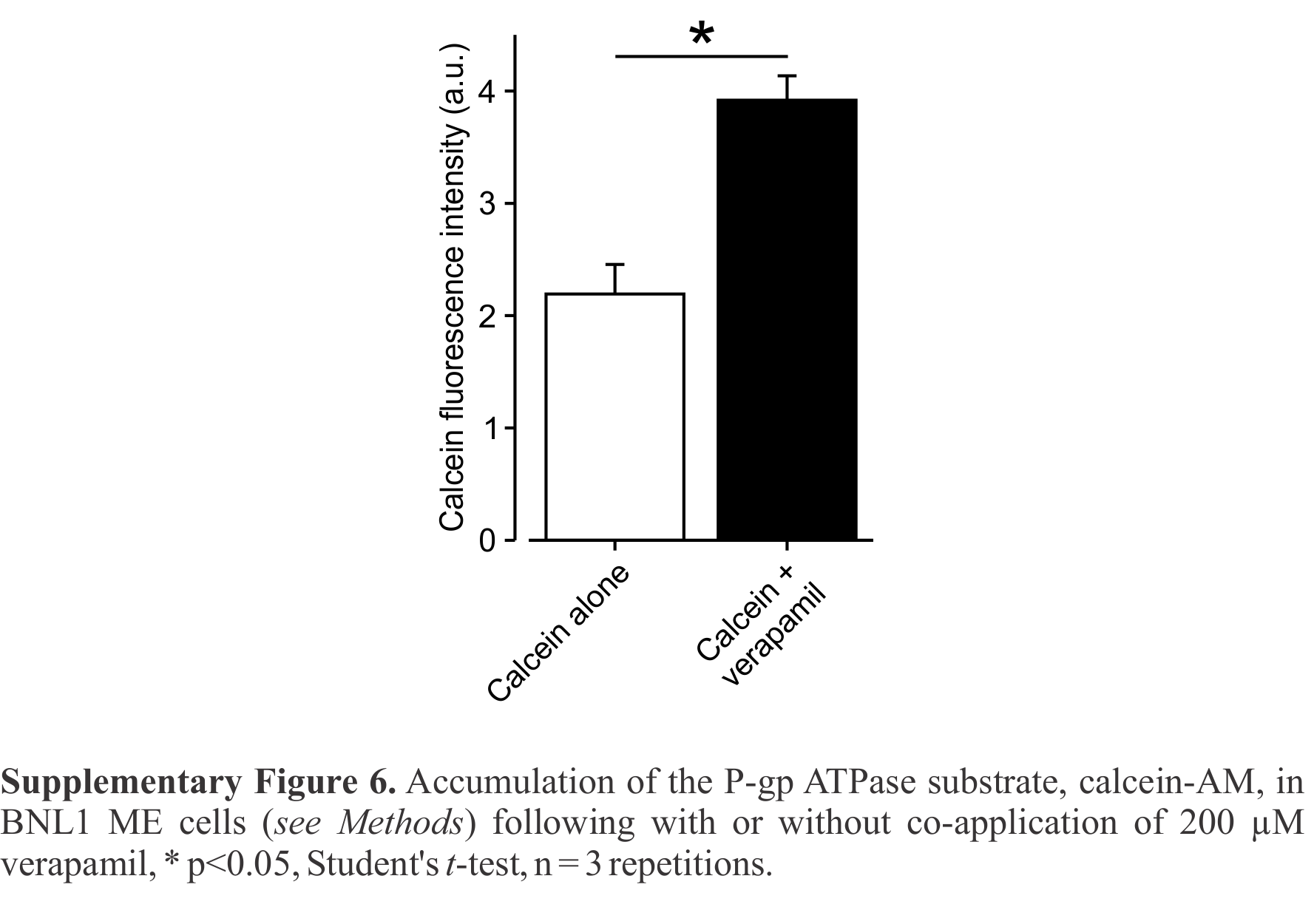

Supplement: Supplementary file 6 [file Image_6.tif]

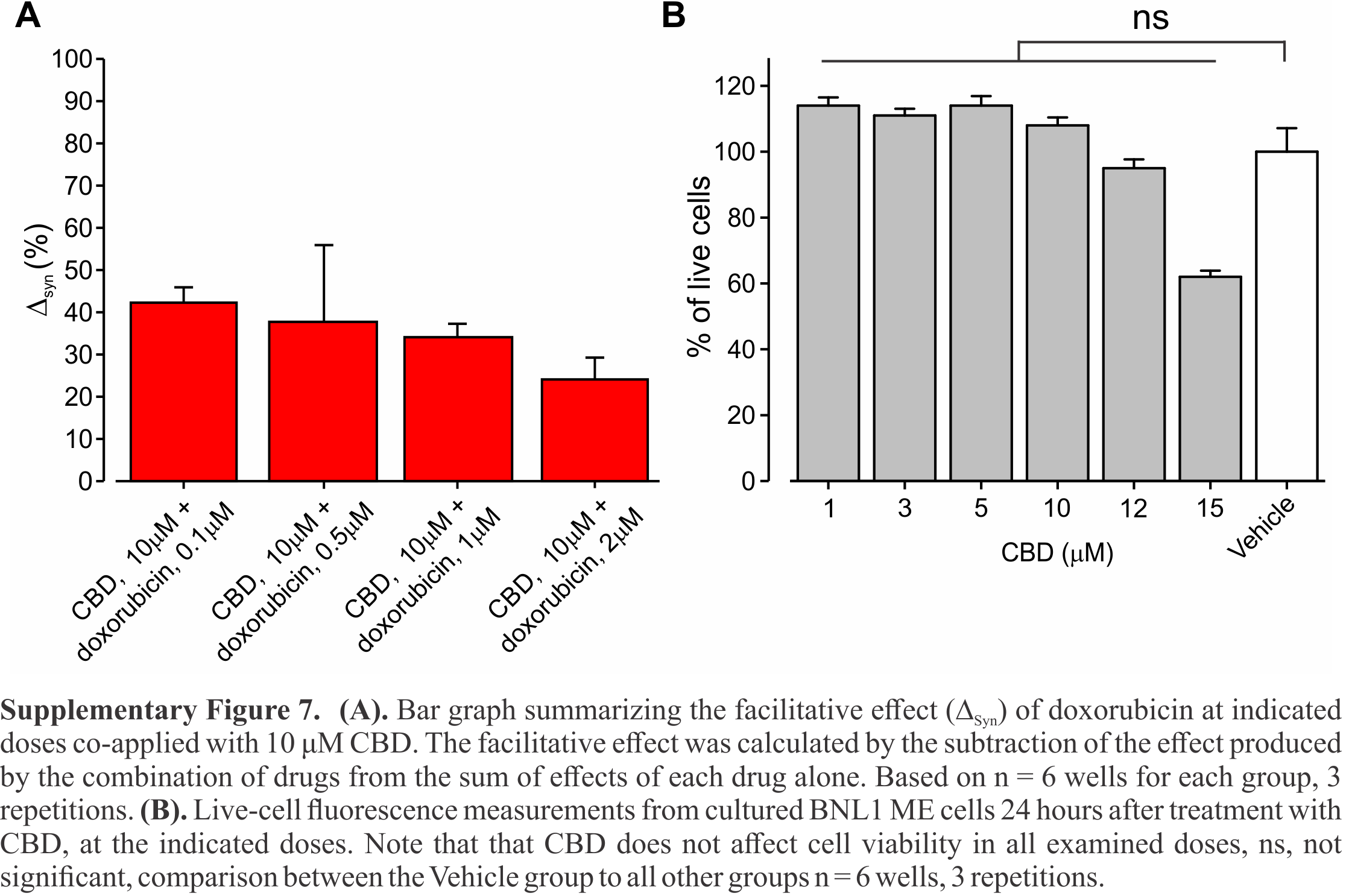

Supplement: Supplementary file 7 [file Image_7.tif]
